# Sound offset responses become highly informative in the auditory cortex

**DOI:** 10.1113/JP288416

**Published:** 2025-09-24

**Authors:** Charly Lamothe, Sophie Bagur, Etienne Gosselin, Brice Bathellier

**Affiliations:** ^1^ Institut Pasteur, AP‐HP, INSERM, CNRS, Fondation Pour l'Audition, Institut de l'Audition, IHU reConnect Université Paris Cité Paris France; ^2^ Brain Plasticity Unit, CNRS, ESPCI‐Paris PSL Research University Paris France

**Keywords:** auditory pathways, offset responses, population decoding, two‐photon imaging

## Abstract

**Abstract:**

The entire auditory system downstream of the cochlea features pronounced offset responses that follow the termination of sounds. Because of their ubiquity, it remains an unsolved question whether offset responses are generated early in the auditory system and subsequently propagated downstream or recomputed at each processing stage. Here, we analysed large‐scale sound response datasets acquired in the cochlear nucleus, inferior colliculus, medial geniculate nucleus and auditory cortex of awake mice. All brain regions showed a significant proportion of offset responses, often combined with onset and sustained responses in the same neuron. However, using population activity decoders, we observed that neural representations after the sound offset show a three‐fold increase in sound encoding accuracy in the cortex relative to subcortical areas. This result indicates that cortical offsets encode a more precise short‐term memory of the elapsed sound than subcortical offsets and that they probably result from specific computational steps.

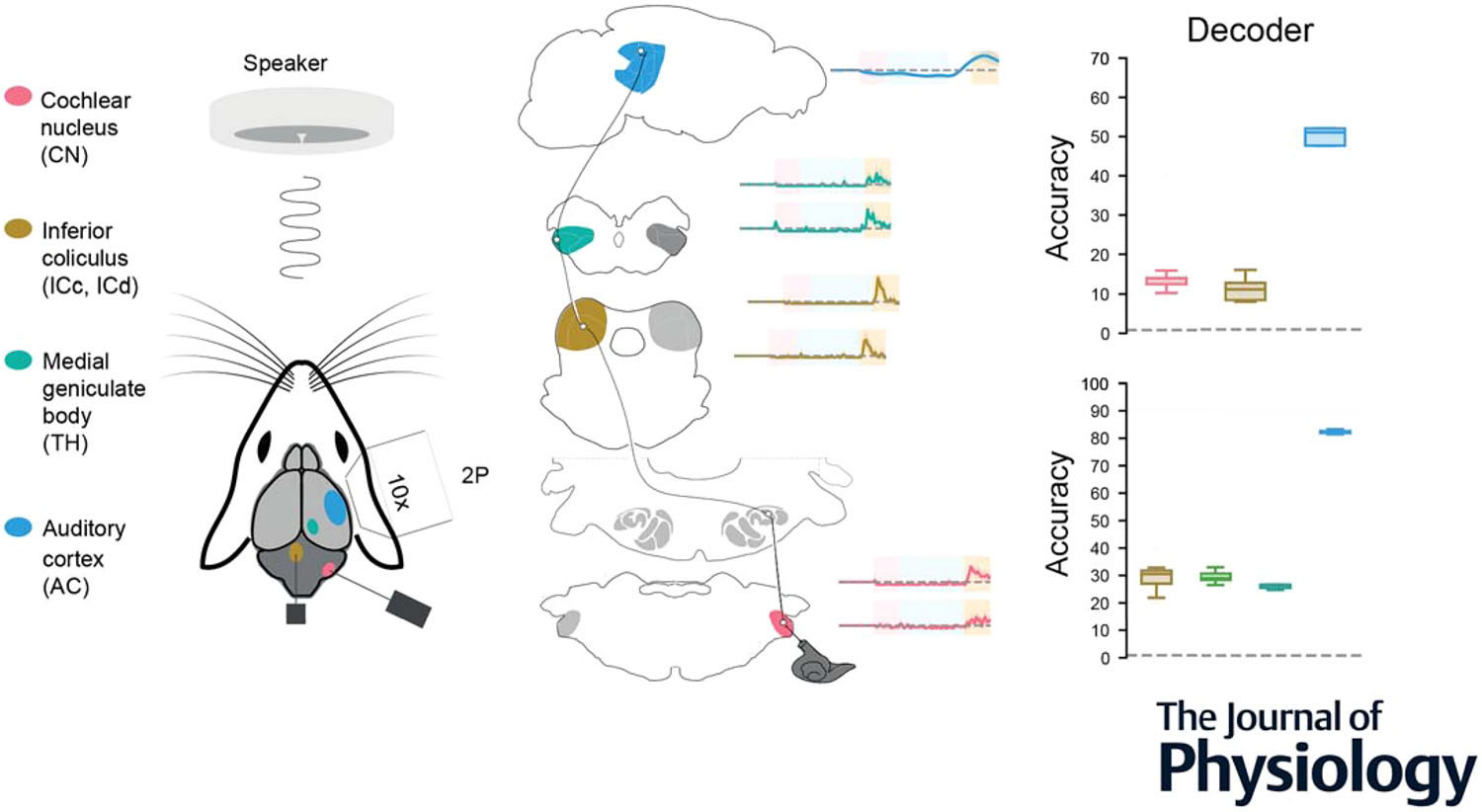

**Key points:**

- Offset responses are found throughout the central auditory system.
- Offset responses are often combined with sustained and onset responses at all central auditory system stages.
- Offset responses provide richer information about elapsed sounds in the auditory cortex.

## Graphical abstract

**Abstract figure legend** Using large datasets of neural responses to diverse sounds recorded across four stages of the mouse auditory system (depicted and colour‐coded on the left), offset responses occurring after the termination of each sound (time window indicated by the orange shaded area superimposed with activity traces) were characterized and it was found using neural population activity classifiers (right plots, colour‐coded as shown on the left) that offset responses are more informative about the sound identity in the auditory cortex than in upstream auditory regions.

## Introduction

Sensory processing is a dynamic phenomenon with a high sensitivity to temporal variations of the stimulus at different time scales. This is particularly acute in the auditory system, which processes complex temporal signals to extract a large range of meaningful features. Response dynamics in the auditory system include salient transient responses at the beginning and at the termination of sounds, often termed sound onset and offset, respectively (Eggermont, 2015; Kopp‐Scheinpflug et al., 2018). Transient onset responses are already present in the auditory nerve, where they mainly reflect adaptation, and are then observed throughout the auditory system. By contrast, no strong elevation of the firing rate is observed in the auditory nerve at sound termination, although some studies report weak inhibitory and excitatory offset responses (Grinnell, 1973; Scheidt et al., 2010; Westerman & Smith, 1984; Yin et al., 2019). Clear excitatory offset responses emerge in the cochlear nucleus (CN) (Kopp‐Scheinpflug et al., 2018; Suga, 1964) and are subsequently present in all downstream stages of the main auditory system. Offset responses are considered to be important to signal sound termination (Anderson & Linden, 2016; Li et al., 2021; Solyga & Barkat, 2021) and gap detection (Awwad et al., 2020), or more generally any abrupt decrease of the sound intensity, which are part of the amplitude modulation features that the brain uses to interpret natural sounds (Bregman, 1994; Eggermont, 2015; Kuwada & Batra, 1999). Offset responses are also recruited by predictive processes, at least in the cortex, to signal omissions of expected gaps (Awwad et al., 2023). However, beyond this widely accepted but very general concept, it remains unclear what information is specifically contained in offset responses and if this information is equivalent at all stages of the auditory system. On the one hand, offset responses could be an unspecific termination signal, independent of the sound that has just ended. On the other hand, offset responses could be a highly specific signal that reflects not only the termination, but also the identity of the sound. For pure tones, frequency specificity of offset responses has long been established at diverse stages (Kasai et al., 2012; Kopp‐Scheinpflug et al., 2018; Xie et al., 2007). However, this is insufficient to assess whether the offset response precisely encodes the identity of the elapsed sound because this identity depends on complex combinations of frequency and temporal features which cannot be probed with pure tones. To determine the level of acoustic information conveyed by offset responses, we analysed sound‐driven activity from several thousand neurons in two recent datasets produced by our laboratory and freely available online. These two datasets contain neural recordings at the four stages of the auditory system from the CN to the auditory cortex (AC). We observed offset responses at all considered stages with similar properties at the single‐cell level. Strikingly, when decoding the identity of elapsed sounds based on neural population responses derived from offset responses, we observed a much better performance in the AC than in subcortical regions. This marked difference suggests that cortical processing not only signals sound termination, but also constructs a rich, transiently maintained representation of the elapsed sound at sound offset.

## Methods

### Ethical approval

All experimental and surgical procedures were carried out in accordance with the European guidelines for animal experiments and were validated by the French Ethical Committee #89 (authorization APAFIS#27040‐2020090316536717 v1). All experimental and surgical procedures strictly comply with policies of *The Journal of Physiology* regarding animal experiments, including housing, anaesthesia and terminal procedures as described below.

### Datasets

All data used in this study are derived from two publicly available datasets summarized in Table 1. Dataset 2 was described more extensively in a published article (Bagur et al., 2025). Dataset 1 is described in a parallel pre‐print manuscript (Gosselin et al., 2025).

**Table 1:** Summary of the datasets

	Method	*N* animals	*N* session	*N* neurons	*N* responsive neurons	Soundset
*Dataset 1*						
CN	Electrophysiology (1 × 384)	8	21	2265	1421 (63%)	119 sounds (pure tones, chords, up/down ramps, up/down frequency sweeps, white noise)
IC‐central	Electrophysiology (1 × 384)	9	15	1197	551 (46%)	
AC	Two‐photon calcium imaging	5	15	14 055	4217 (30%)	
*Dataset 2*						
IC‐central	Electrophysiology (1 × 32)	11	30	563	442 (78%)	112 sounds (pure tones, up/down ramps, up/down frequency sweeps)
IC‐dorsal	Two‐photon calcium imaging	30	101	15 312	5936 (39%)	
TH	Electrophysiology (4 × 8)	10	33	498	484 (97%)	
AC	Two‐photon calcium imaging	7	60	60 822	19 414 (32%)	

### Subjects and ethical authorization

All mice used for the production of the datasets were 10–16‐week‐old male C57Bl6J mice (26–30 g) obtained from Janvier Labs (Le Genest‐Saint‐Isle, France) that had not undergone any other procedures. Mice were group‐housed (two to four per cage) before and after surgery, had *ad libitum* access to food and water, and enrichment (running wheel, cotton bedding and wooden logs), and were maintained under a 12:12 h light/dark photocycle in controlled humidity and temperature conditions (21–23°C, 45%–55% relative humidity). All experiments were performed during the light phase. The terminal procedure was carbon dioxide inhalation.

### Surgical procedures

Mice were injected with buprenorphine (Vétergesic, Ceva Santé Animale, Libourne, France; 0.05–0.1 mg kg^−1^) 30 min prior to surgery. Surgical procedures were carried out using either i.p. ketamine (Ketasol, Interchemie Werken De Adelaar Eesti AS, Viimsi, Estonia) and medetomidine (Domitor, Orion Pharma, Espoo, Finland) which was antagonized with atipamezole (Antisedan; Orion Pharma, Espoo, Finland) at the end of the surgery or 1.5% isoflurane delivered via a mask. After induction, mice were kept on a thermal blanket during the whole procedure and their eyes were protected with Ocrygel (Le Vet. Beheer, Tv Oudewater, Netherlands). Lidocaine was injected under the skin of the skull 5 min prior to incision.

For electrophysiology recordings, the skull was exposed at the relevant location for ulterior craniotomy: above the inferior colliculus (IC), above the cortex dorsal to the medial geniculate nucleus (TH) or above the dorsal cerebellum for the CN. A well was formed around it using dental cement in order to retain saline solution during recordings and the head post was fixed to the skull using cyanolit glue and dental cement. To protect the skull, the well was filled with a waterproof silicone elastomer (Kwik‐Cast; World Precision Instruments, Sarasota, FL, USA) that could be removed prior to recording. The head post was fixed to the skull using cyanolit glue and dental cement (Ortho‐Jet; Lang, Wheeling, IL, USA).

For calcium imaging, craniotomies of 3 mm (IC) or 5 mm (AC) were performed above the IC or the AC. Injections of 150 nL of AAV1.Syn.GCaMP6s.WPRE (Vector Core, Philadelphia, PA, USA; 10^13^ viral particles mL^−1^; used pure for TH and diluted 30× for AC and IC) were made at 30 nL min^−1^ with pulled glass pipettes at a depth of 500 µm and spaced every 500 µm to cover the large surface of the IC or AC. The craniotomy was sealed with a circular glass coverslip. The coverslip and head post were fixed to the skull using cyanolit glue and dental cement (Ortho‐Jet; Lang).

After surgery, mice received a subcutaneous injection of 30% glucose and metacam (1 mg kg^−1^). Mice were subsequently housed for 1 week with metacam delivered via drinking water or dietgel (ClearH20). Mice were given 1 week to recover from surgery without any manipulation. Then, for 4 days before recording, mice were habituated to head restraint for increasing periods of time (30 min to 2 h). For electrophysiological experiments, the day before recording, animals were briefly anesthetized using isoflurane anaesthesia (2%) to perform craniotomy and durectomy for electrode descent.

### Electrophysiological recordings

#### Dataset 1 (CN)

The awake mouse was head‐fixed and Neuropixels 1.0 probes (384 channels) were inserted through the cerebellum at a 38°–40° angle in the sagittal plane, targeting the contralateral CN, vertically targeting the IC, or both. Electrode angle and entry point were defined relative to the initial head‐post placement. Fine‐tuning of these targeting parameters was progressively obtained through repeated penetrations based on time‐locked responses to sounds easily detectable during probe insertion. Data were sampled at 30 kHz using a NI‐PXI chassis (National Instruments, Austin, TX, USA) and the SpikeGLX (https://github.com/billkarsh/SpikeGLX) acquisition software.

#### Dataset 2 (IC, TH)

The awake mouse was head‐fixed and a Neuronexus 1 × 32 linear probe (IC) or 4 × 8 comb probe (TH) was inserted. Data were sampled at 20 kHz using an RHD2000 amplifier board (Intan Technologies, Los Angeles, CA, USA).

Recording was started 15–20 min after the electrode position was locked to allow the brain tissue to stabilize and minimize movements of neurons in the first part of the recording. Recordings were performed using warmed saline filling the cyanolit glue well and in contact with the reference electrode. After each recording the well was amply flushed and then refilled with Kwik‐Cast.

For *post hoc* histological verification of the electrode track using fluorescent dye, the electrodes were dipped in diI, diO or diD (Vybrant Multicolor Cell‐Labelling Kit; Thermo Fisher Scientific, Waltham, MA, USA) prior to insertion.

### Two‐photon calcium imaging

#### Dataset 1 (AC)

Imaging was performed using an acousto‐optic two‐photon microscope (Karthala System, Orsay, France) with a pulsed laser (Insight, Spectra‐Physics, Santa Clara, CA, USA) set at 900 nm. We used a 16× objective (N16XLWD‐PF; Nikon, Tokyo, Japan) to acquire stacks of four images at 22.9 Hz from four planes interleaved by 50 µm in a field of view of 478 × 478 µm.

#### Dataset 2 (AC and IC)

Imaging was performed using a two‐photon microscope (Femtonics, Budapest, Hungary) equipped with an 8 kHz resonant scanner combined with a pulsed laser (MaiTai‐DS; Spectra‐Physics) set at 900 nm. We used a 10× objective (XLPLN10XSVMP; Olympus, Tokyo, Japan), which provided a field of view of up to 1 × 1 mm. For AC, a 1 × 1mm field of view was used. For IC, the field of view was adjusted to the size of the structure (∼0.5 × 0.5 mm). Images were acquired at 31.5 Hz.

### Sound set and experimental protocol

#### Dataset 1

Sounds were generated with Python (The Python Software Foundation, Wilmington, DE, USA) and were delivered at 192 kHz with Matlab (The Mathworks, Natick, MA), using a NI‐PCI‐6221 card (National Instruments) driven by a custom protocol using the Matlab Data Acquisition toolbox and feeding an amplified free‐field loudspeaker (SA1 and MF1‐S; Tucker‐Davis Technologies, Alachua, FL, USA) positioned in front of the mouse, 10–15 cm from the mouse's ear. Sound intensity was cosine‐ramped over 10 ms at the onset and offset to avoid spectral splatter. The head‐fixed mouse was isolated from external noise sources by sound‐proof boxes (custom‐made; Decibel France, Miribel, France) providing 30 dB attenuation above 1 kHz. Sound pressure levels were computed as the root mean square. During a recording session, 307 sounds were repeated 12 times and played in a random order with a 1 s interval between sound onsets in 123 blocks of 30 sounds (total duration ∼80 min). The 307 sounds included simple and more complex sounds. Because of the strong time‐varying nature of the complex sounds we focused the analysis of offset responses on a set of 152 simpler sounds whose duration was 500 ms. These included 28 pure tones: pure tones at 14 frequencies logarithmically spaced between 2 and 60 kHz at 50 dB SPL and 70 dB SPL. 26 ramps: linearly ramped sounds, increasing and decreasing in intensity, at the same frequencies as the 13 pure tones between 2 kHz and 50 kHz between 50 dB SPL and 70 dB SPL. 48 chords: summation of two to four 70 dB pure tones from low (11 sounds), medium (11 sounds), high (11 sounds) frequency groups, broadly sampled frequencies (10 sounds) and harmonically arranged frequencies (five sounds). 20 chirps: up‐ and down‐ frequency sweeps of different durations between 25 ms and 400 ms at 6–12 kHz, 50 dB SPL (10 sounds) or different frequency content between 4 kHz and 50 kHz at 50 dB SPL in 500 ms (10 sounds). 30 coloured noises (WN): broadband noises at 50 dB SPL, 70 dB SPL or up‐/down‐ramped (six sounds), filtered noises at different bandwidths between 2 kHz and 80 kHz (14 sounds), and summation of two 1 kHz bandwidth filtered noises up‐ and down‐ramped between 50 dB SPL and 70 dB SPL, matching frequencies of a subset of chords (10 sounds). 48 amplitude‐modulated sounds (AM): sinusoidally amplitude modulated sounds at six different modulation frequencies between 4 Hz and 160 Hz and eight carrier frequency contents (two pure frequencies, five sums of frequencies matching chords and one broadband noise). Out of the 152 sounds, 33 produced unreliable population responses (mean correlation across time‐averaged single‐trial population vectors <0.1) in at least one of the three recorded areas. These sounds were excluded from the final analysis, restricted to 119 sounds, to focus on robust responses. We verified that this had no effect on our conclusions.

#### Dataset 2

Sounds were generated with Matlab (The Mathworks) and were delivered at 192 kHz with a NI‐PCI‐6221 card (National Instruments) driven by the software Elphy (G. Sadoc, UNIC, France) and feeding an amplified free‐field loudspeaker (SA1 and MF1‐S; Tucker‐Davis Technologies, Alachua, FL, USA) positioned 15–20 cm from the mouse's ear. Sound intensity was cosine‐ramped over 10 ms at the onset and offset to avoid spectral splatter. The head‐fixed mouse was isolated from external noise sources by sound‐proof boxes (custom‐made by Femtonics, Budapest, Hungary or Decibel France, Miribel, France) providing 30 dB attenuation above 1 kHz. Sounds were calibrated in intensity at the location of the mouse's ear using a probe microphone (type 4939‐L‐002; Bruel & Kjaer, Virum, Denmark). For two‐photon calcium imaging, the resonant scanner generated a harmonic background noise at 8 kHz (intensity at the mouse's ear, 45 dB SPL). During a recording session, each sound was presented 15 times in random order.

The set of sounds consisted of 112 sounds (Fig. 1) which were all 500 ms long. 26 pure tones: pure tones at 11 frequencies logarithmically spaced between 4 kHz and 37 kHz at 50, 70 and 80 dB SPL. 42 ramps: linearly ramped sounds, increasing and decreasing in intensity, at various frequencies and combinations (chords) of frequencies. 44 chirps: up‐ and down‐ frequency sweeps with various start and stop frequencies between 4 kHz and 24 kHz at 50 dB and 80 dB. These sounds were played in pseudo‐random order together with 28 other sounds (shorter frequency ramps and long amplitude‐modulated sounds) (Bagur et al., 2025), which were excluded from the present analysis.

**Figure 1:**
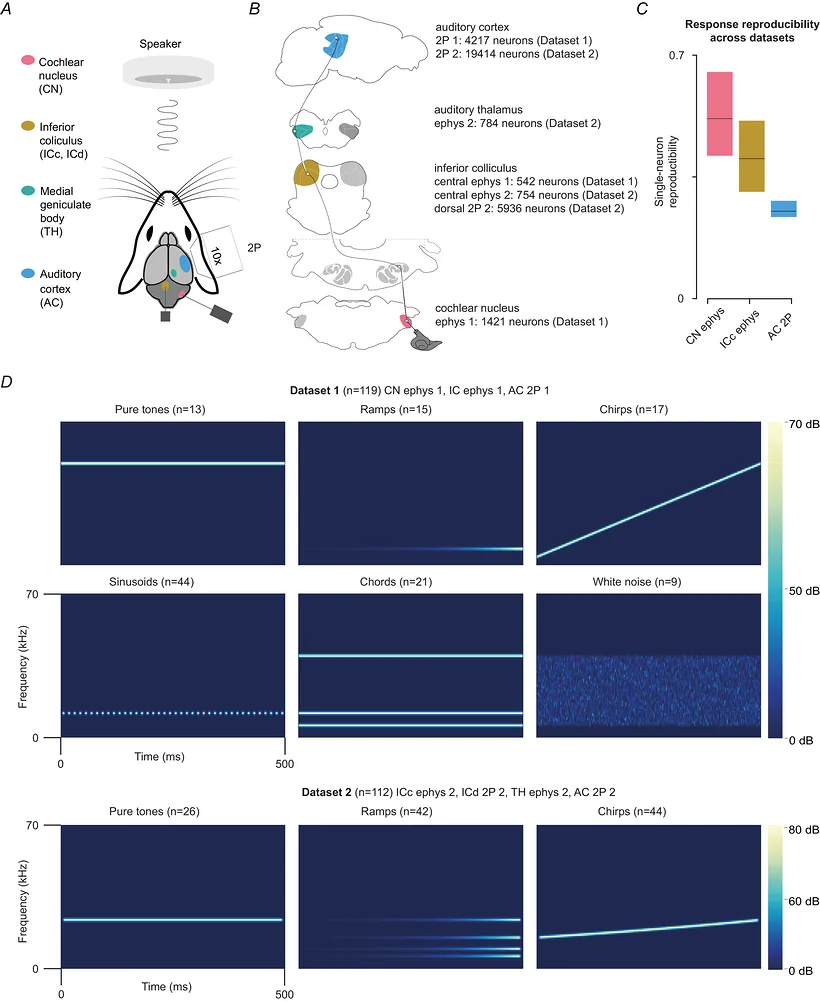
Description of the two multi‐area auditory response datasets *A*, schematic summarizing the recording methods (extracellular multi‐electrode electrophysiology: ‘ephys’, and two‐photon calcium imaging: ‘2P’) and the four recorded areas. *B*, summary of the number of putative neurons recorded in each region for each dataset. *C*, response reproducibility across datasets. *D*, sample spectrograms for each sound type played in each of the two datasets.

### Data preprocessing

#### Dataset 1

Motion artefacts, regions of interest selection, and the signal extraction were carried out with the standard pipeline of the Python‐based version of Suite2p (Pachitariu et al., 2017). For each region of interest, the mean fluorescence signal *F*(*t*) was extracted together with the local neuropil signal *F*_np_(*t*). Then, 70% of the neuropil signal was subtracted from the neuron signal to limit neuropil contamination. Baseline fluorescence *F*_0_ was calculated with a sliding window computing the third percentile of a Gaussian‐filtered trace over the imaging blocks. Fluorescence variations were then computed as *f*(*t*) = Δ*F/F* = (*F*(*t*) – *F*_0_)/*F*_0_. An estimate of firing rate variations r(t) was then obtained by linear temporal deconvolution of f(t): *r*(*t*) = *f'*(*t*) + *f*(*t*)*/τ*, *f'*(*t*) being the first derivative of *f*(*t*) and τ = 2s, the estimated decay of the GCAMP6s fluorescent transients. This simple method efficiently corrects the strong discrepancy between fluorescence and firing rate time courses because of the slow decay of spike‐triggered calcium transients. It does not correct for the rise time of GCAMP6s, leading to remnant low‐pass filtering of the firing rate estimate and a delay of ∼100 ms between the firing rate peaks and the peaks of the deconvolved signal. Finally, response traces were smoothed with a Gaussian filter (σ = 31 ms). Single‐trial sound responses were extracted (0.2 s before up to 0.8 s after sound onset) and the average activity over the prestimulus period (0.2–0.02 s before sound onset) was subtracted for each trial.

For electrophysiology, raw data were band‐pass filtered (300–6000 Hz) and channels from the electrode tip (corresponding to the CN region) were selected using SpikeInterface (https://github.com/SpikeInterface) (Buccino et al., 2020). Isolated clusters were identified using Kilosort 2.5 (Pachitariu et al., 2024) followed by manual curation based on the interspike‐interval histogram and the inspection of the spike waveform using Phy (https://github.com/cortex-lab/phy). Canonical spike sorting was first applied with common parameters throughout the whole recording, attempting to optimize the spike detection and assignment to clusters. The measure of drift throughout the recording, computed with Kilosort 2.5 (Pachitariu et al., 2024), showed minimal slow drift throughout the recording, except for some experiments in the first 10 min. Because of the high spiking activity in the CN, optimal spike sorting was achieved using lower Kilosort 2.5 parameters (detection threshold = 6, clustering threshold = 6, matching thresholds = [9,4]) in this region than for the IC (detection threshold = 8, clustering threshold = 8, and matching thresholds = [10,4]). After manual curation, single‐trial sound responses were extracted (0.2 s before up to 0.8 s after sound onset) as a histogram of 1ms time bin and the average activity over the prestimulus period (0.2–0 s before sound onset) was subtracted for each trial. Based on histology, we identified a number of units whose location on the Neuropixel probe was not compatible with a localization in the CN or IC.

#### Dataset 2

For calcium imaging, regions of interest corresponding to putative neurons (AC 2P and IC) or axons and boutons (TH ephys) were identified by using Autocell (Deneux et al., 2016) (https://github.com/thomasdeneux/Autocell). Briefly, each frame of the recording was corrected for horizontal motion using rigid body registration. This step was visually controlled and all sessions with visible z motion were eliminated. A hierarchical clustering algorithm, based on pixel covariance over time, agglomerated pixels up to a user‐selected number of clusters corresponding to regions of the size of neurons of axons. Clusters were automatically filtered according to size and shape criteria. This step was controlled by a detailed visual inspection of selected regions of interest (ROIs) during which ROIs without visually identifiable cell body shape were discarded.

For each region of interest, the mean fluorescence signal *F*(*t*) was extracted together with the local neuropil signal *F_np_* (*t*). Then, 70% of the neuropil signal was subtracted from the neuron signal to limit neuropil contamination. Baseline fluorescence *F*_0_ was calculated with a sliding window computing the third percentile of a Gaussian‐filtered trace over the imaging blocks. Fluorescence variations were then computed as *f*(*t*)* = ΔF/F = *(*F*(*t*)* – F_0_*)*/F_0_*. An estimate of firing rate variations *r*(*t*) was then obtained by linear temporal deconvolution of f(*t*)*: r*(*t*)* = f'*(*t*) + *f*(*t*)*/τ*, *f'*(*t*) being the first derivative of *f*(*t*) and τ = 2s, the estimated decay of the GCAMP6s fluorescent transients. This simple method efficiently corrects the strong discrepancy between fluorescence and firing rate time courses because of the slow decay of spike‐triggered calcium transients. It does not correct for the rise time of GCAMP6s, leading to remnant low‐pass filtering of the firing rate estimate and a delay of ∼100 ms between the firing rate peaks and the peaks of the deconvolved signal. Finally, response traces were smoothed with a Gaussian filter (σ = 31 ms).

Electrophysiological signals were high‐pass filtered and spike sorting was performed using the CortexLab suite (https://github.com/cortex‐lab; UCL, London, England). Single‐unit clusters were identified using Kilosort 2.5 (Pachitariu et al., 2024), followed by manual corrections based on the interspike‐interval histogram and the inspection of the spike waveform using Phy (https://github.com/cortex-lab/phy).

Both for imaging and electrophysiology data, single‐trial sound responses were extracted (0.5 s before and 1 s after sound onset) and the average activity over the prestimulus period (0.5–0 s before sound onset) was subtracted for each trial.

### Reproducibility index and cell selection

To quantify the noise levels in the data, we calculated the mean inter‐trial correlation across all pairs of trials. The single neuron reproducibility is then defined for each neuron as the average of the inter‐trial correlation for that neuron's response to all sounds presented in the experiment. ROIs or single units with reproducibility below 0.12 were classified as non‐responsive and were excluded from all analyses. The number of responsive units and the corresponding fraction of the total number of units/ROIs are given in Table 1.

### Population activity decoding

To decode stimulus identity from neural activity, we evaluated performance across different response windows, including onset, sustained, offset, and full response periods. For each window, neural responses from all neurons in each considered dataset were extracted and concatenated into a virtual population vector. In order to avoid statistical threshold effects, the selection of offset responses based on single neuron responses was not used in the population decoding approach. These vectors were constructed by averaging neural responses across the analysis time bin (onset, sustained, offset) except for the measurement of ceiling information. For the latter analysis, 65 time bins were defined from 0 to 150 ms after stimulus onset (650 ms in total), a population vector was constructed for each time bin, and vectors from all time bins were concatenated into a vector summarizing the full spatio‐temporal activity time‐course of the population.

Next, the neural responses from each window were used as input to a stratified five‐fold cross‐validation logistic regression decoder. Stratified cross‐validation ensured that the class distributions were preserved in each fold, addressing any class imbalances. During each fold, the training and test sets were normalized by subtracting the training set mean. This approach allowed us to assess the fraction of correct sound identity predictions based on the neural population's activity during each response window. The decoding performance was evaluated using balanced accuracy.

### Response profile identification

To identify onset and sustained response for each neuron and sound, we used a non‐parametric, paired Wilcoxon signed‐rank test comparing baseline activity to onset or sustained activity respectively. The alpha value of the test was 0.05, implying that the proportion of neuron‐pairs with significant responses has to be above 5% to exceed the maximum chance level. For offset response detection, we used two non independent paired Wilcoxon signed‐rank tests comparing offset activity to baseline and sustained activity, respectively, with an alpha value of 0.05 for each test. Because the single‐trial measurements of baseline and sustained activity are done at a short time interval, they are not statistically independent. Therefore, the false discovery rate (FDR) is not the product of alpha values as in the case of two independent tests, but is probably higher. To be conservative, we used the alpha value of one signed‐rank test (0.05, same for both tests), as an estimate of the false detection rate (FDR, i.e. chance level) for offset responses. Significant responses were classified as positive or negative based on the sign of the time‐averaged baseline‐subtracted activity over the analysis time bin. To estimate the fraction of neurons with at least one offset response (independent of its sign), we used the Benjamini–Hochberg procedure on each neuron to correct FDR across the 119 (dataset 1) and 112 (dataset 2) signed‐rank tests performed on each sound. Therefore, the chance level for the fraction of neurons responding with at least one offset is also 5%. The degrees of freedom of the signed‐rank test correspond to the number of sound repetitions: *N* = 12 for dataset 1, *N* = 15 for dataset 2.

### Linear decomposition of responses

To separate the onset, sustained, and offset components, we used a linear model to approximate the response function. The linear model is a sum of three regressors (on, off and sustained) with fixed shape but whose amplitude is fit to the neural response. Because we do not know with sufficient precision the underlying biophysical mechanisms resulting in the response shape that moreover probably vary between neurons, we empirically designed the shape of the three regressors to resemble the data:

$$\mathrm{ON}\;\left(t\right)=\exp\left(-\frac{t}{\tau_{\mathrm{decay}}}\right)\;-\exp\left(-\frac{t}{\tau_{\mathrm{rise}}}\right)$$

with $\tau_{\mathrm{decay}}=0.075\;s$ and $\tau_{\mathrm{rise}}=0.05\;s$

$$\mathrm{SUST}\;\left(t\right)={\mathrm{sustcomp}}_{\mathrm{rise}}\left(t\right)-{\mathrm{sustcomp}}_{\mathrm{decay}}\left(t\right)$$

$${\mathrm{sustcomp}}_{\mathrm{rise}}\;\left(t\right)=1-\exp\left(-\frac{t}{\tau_{\mathrm{rise}}}\right)$$

$${\mathrm{sustcomp}}_{\mathrm{rise}}\;\left(t\right)=\left\{\begin{array}{c} 1-exp\left(-\frac{t}{\tau_{\mathrm{decay}}}\right)\mathrm{if}\;t>0.5\\ 0\;\mathrm{if}\;t<0.5 \end{array}\right.\;\;$$

with $\tau_{\mathrm{decay}}=0.075\;s$ and $\tau_{\mathrm{rise}}=0.05\;s$

$$\mathrm{OFF}\;\left(t\right)=\left\{\begin{array}{c} \exp\left(-\frac{t}{\tau_{\mathrm{decay}}}\right)-exp\left(-\frac{t}{\tau_{\mathrm{rise}}}\right)\mathrm{if}\;t>0.5\\ 0\;\mathrm{if}\;t<0.5 \end{array}\right.$$

with $\tau_{\mathrm{decay}}$ = 0.075|0.05|0.1 s and $\tau_{\mathrm{rise}}$= 0.1|0.1|0.12 s

After defining three regressors, the neural response is defined by this linear equation:

$$\mathrm{Resp}\;\left(t\right)=\alpha_{\mathrm{ON}}\;\mathrm{ON}\left(t\right)+\alpha_{\mathrm{SUST}}\;\mathrm{SUST}\left(t\right)+\alpha_{\mathrm{OFF}}\;\mathrm{OFF}\left(t\right)$$

The amplitudes (α_ON_, α_SUST_ and α_OFF_) can be directly calculated (here, using Matlab's backslash function). Given the importance of correctly fitting the offset response, we performed the fitting procedure with three different time constants and retained the best for further analysis. This fitting procedure was performed for all neurons, all sounds and every trial, and the amplitudes were then used to perform classification as previously described.

## Results

### Offset responses are prominent at all stages of the auditory system

This study is based on two datasets shared in open source repositories (Bagur & Bathellier, 2024; Gosselin, 2024). The first dataset (Fig. 1*A* and *B*) (Gosselin, 2024) is more extensively described in a parallel study (Gosselin et al., 2025), which does not focus on offset responses. It includes Neuropixel recordings (https://www.neuropixels.org) in awake mice that sampled 1421 putative neurons (single units) in the CN (CN, ephys 1) and 551 single units in the IC (IC, ephys 1). This dataset also includes two‐photon calcium imaging of 4217 neurons in the AC of awake mice (AC, 2P 2). Notably, response variability was greater in the AC dataset, as measured by the correlation of single‐trial responses to the same sound, averaged across all neurons and stimuli (Fig. 1*C*). For all neurons in this dataset, we collected responses to 307 different sounds, each repeated 12 times. For this study, we kept 119 sounds that are all 500 ms long and contain different spectral (pure tones, frequency chords and white noise) and temporal (frequency sweeps, rhythmic and oriented amplitude modulations) features (Fig. 1*D*). The second dataset (Fig. 1*A* and *B*) (Bagur & Bathellier, 2024) was already used to describe population representations of simple time‐varying sounds (Bagur et al., 2025). It includes silicon probe recordings in awake mice that sampled 442 putative neurons (single units) in the IC (IC, ephys 2) and 484 single units in the TH (TH, ephys 2). This dataset also includes two‐photon calcium imaging of 5936 neurons in the dorsal IC (IC, 2P 2) and 19 414 neurons in the AC of awake mice (AC, 2P 2). As in dataset 1, larger response variability is observed in two‐photon imaging data as reported in (Bagur et al., 2025) with the same measure as in Fig. 1*C*. For all sampled neurons of this dataset, we collected responses to 140 different sounds, each repeated 15 times. For this study, we kept 112 sounds that are all 500 ms long and contain different spectral (pure tones and frequency chords) and temporal (frequency sweeps and oriented amplitude modulations) features (Fig. 1*D*). For extracellular electrophysiology data, single units were identified with Kilosort 2.5 (Pachitariu et al., 2024), combined with manual curation in Phy. For calcium imaging data, motion correction and neuron segmentation were performed with freely available tools (see Methods). The data were then temporally deconvolved using a simple linear algorithm, allowing for a temporal precision of about 3 Hz (Bagur et al., 2025). This is sufficient to separately identify onset and offset responses for the 500 ms sounds selected in this study (Fig. 2).

**Figure 2:**
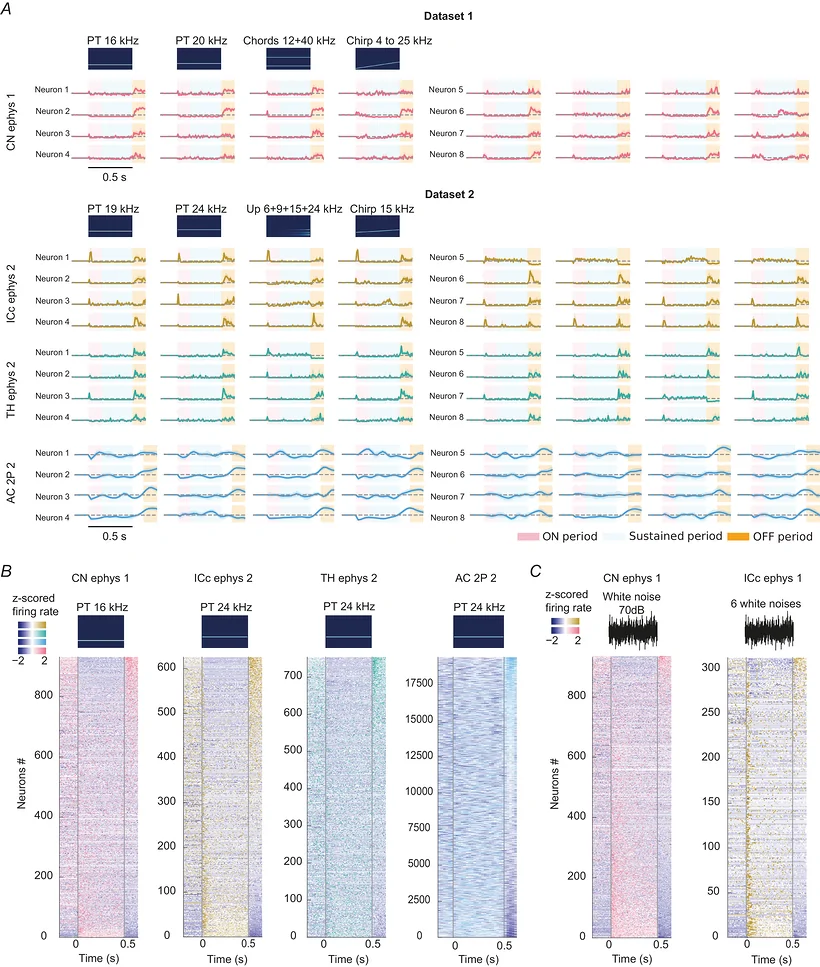
Offset responses are prominent at all stages of the auditory system *A*, sample responses of three selected neurons in each of the recorded areas for six different sounds. Neurons of the cochlear nucleus (CN) come from dataset 1 and neurons of the inferior colliculus (IC), medial geniculate nucleus (TH) and auditory cortex (AC) from dataset 2. *B*, heat map of the responses of all recorded neurons for the areas and datasets shown in (*B*) for one sound (Pure tone at 16 kHz/70 dB SPL and 24 kHz/80 dB SPL). *C*, left: heat map of the responses of responsive neurons to white noise at 70 dB SPL in the cochlear nucleus (CN ephys 1); right: heat map of the responses of responsive neurons to six different white noise sounds (different intensity profiles) in the inferior colliculus (IC ephys 1).

Plotting response traces of sample neurons from all four recorded regions, we observed that offset responses were present at all stages of the central auditory system, consistent with previous observations. Of note, the temporal deconvolution of calcium imaging signals in the two‐photon datasets (cortex and colliculus) removes the effects of the slow decay time constant of calcium dynamics but does not cancel the effects related to the slow rise time constant of GCAMP6s (∼50–100 ms). Therefore, the peak of offset responses in calcium imaging datasets (e.g. Fig. 2*A*) occurred typically 100 ms later than in electrophysiology recordings (Fig. 2*A*). At all stages, offset response duration could be short (few tens of millisecond) or prolonged (>100 ms), indicating that long timescales of activity are not specific to the cortex or to calcium imaging data. This was apparent in both single cell traces (Fig. 2*A*) and heat maps representing all cell responses ordered according to the offset amplitudes (Fig. 2*B*). The latter plots also show that offset responses can be either excitatory or inhibitory (Fig. 2*B*). Offset responses were observed not only for pure tones (Fig. 2*A* and *B*), but also for broad frequency sounds such as white noise (Fig. 2*C*). We also observed that offset responses often co‐occurred with excitatory or inhibitory onset or sustained responses, consistent with various reports (Scholl et al., 2010; Sollini et al., 2018; Xie et al., 2007) (Fig. 2*A*). This indicates, as previously reported (Kopp‐Scheinpflug et al., 2018), that many neurons are not dedicated to offset responses.

### Offset responses are complex at all stages of the auditory system

To determine whether neurons with a mixture of onset, sustained or offset selectivity appear early or late in the auditory system, we systematically identified statistically significant responses in each phase (time bins: baseline −200 to 0 ms, onset 0–150 ms, sustained 150–500 ms, offsets 500–650 ms for electrophysiology and 600–750 ms for 2P‐imaging) (Fig. 3*A*). Significant onset or sustained responses were identified by comparing firing rates during the onset or sustained phases to firing rates during baseline periods using a non‐parametric test (Fig. 3*A*) (see Methods). For offset responses, we used two non independent non‐parametric tests (Fig. 3*A*) to determine whether offset activity was different from both the baseline and the sustained response. For onset, sustained and offset periods, the maximal false discovery rate was 5%, based on the alpha value of the tests (note that the two tests of offset detection being non independent, the overall, conservative false discovery rate is also 5%) (see Methods).

**Figure 3:**
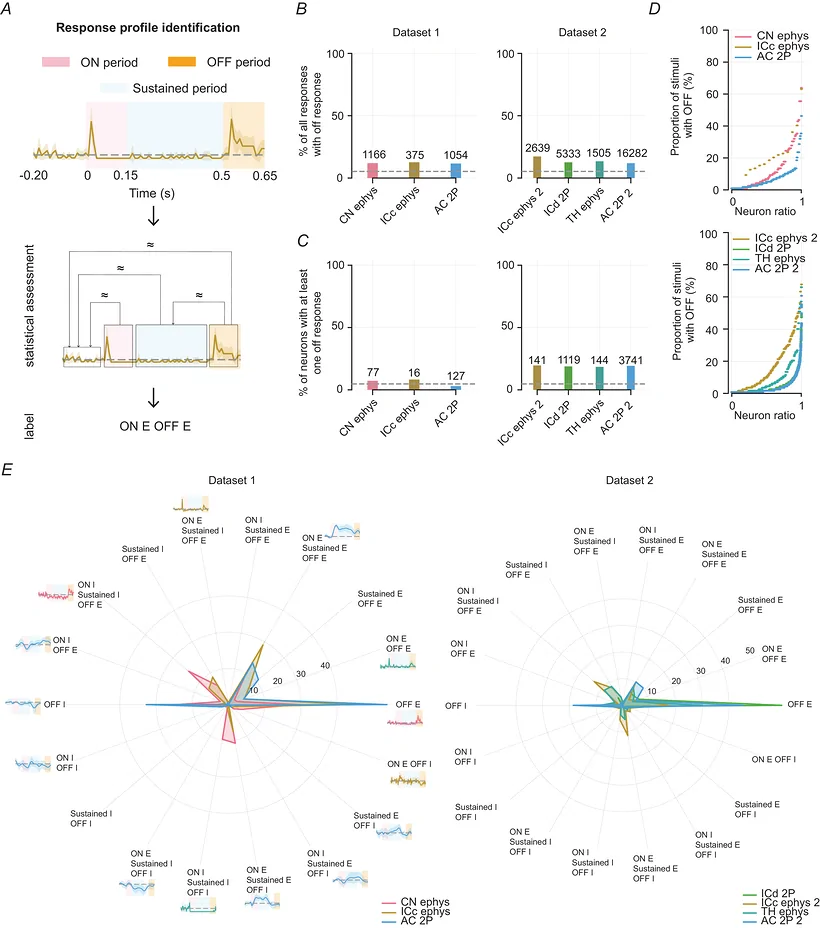
Offset responses are complex at all stages of the auditory system *A*, schematic of the statistical method used to detect onset, sustained and offset responses. *B*, fraction of all pairs of sound responses and neurons displaying a significant change of firing rate at the offset. The grey dotted line indicates chance level (FDR, alpha of 0.05). *C*, fraction of neurons for which at least one significant positive or negative offset response was detected among all tested sounds. The grey dotted line indicates chance level (FDR, alpha of 0.05). *D*, distribution of the fraction of responses with a significant change of firing rate (positive or negative) at the offset for each dataset. *E*, fraction of all pairs of sound responses and neurons corresponding to each possible combination of positive (E) or negative (I) onset (ON), sustained and offset (OFF) responses (18 combinations).

Based on this approach, we first quantified the fraction of all sound responses (number of sounds × number of neurons) that included a significant offset response (positive or negative) at each recorded auditory system stage (Fig. 3*B*). We found this fraction to range from 11.67% to 12.65% in dataset 1 and from 11.91% to 17.31% in dataset 2, which was therefore systematically above the 5% chance level and consistent with the reported sparseness of offset responses. We then estimated the fraction of neurons with at least one offset response using the signed‐rank tests for responses to individual sounds but applying the Benjamini–Hochberg procedure to correct for multiple testing (119 and 112 tests per neuron in datasets 1 and 2, respectively). These estimates are probably conservative as a result of the low number of sound repetitions, which limits the statistical power of this offset‐neuron identification procedure, with presumably many false negative detections. In dataset 1, in which we observed fewer responses (Fig. 3*B*), the fraction of neurons with detected offset is roughly equal to or below the chance level: 7.28% for CN, 8.29% for IC, 3.01% for AC (Fig. 3*C*). In dataset 2, the fraction of neurons with detected offset is clearly above chance level: 18.85% for IC_2P, 19.61% for IC_ephys, 18.44% for TH, 19.27% for AC (Fig. 3*C*). Using the results of the same statistical procedure, including correction for multiple testing, we also plotted the distribution of the number of offset responses per neuron. We observed that less than 50% of the neurons responded at the offset of more than 20 sounds, yet with an even narrower distribution for datasets derived from two‐photon calcium imaging (IC and AC) (Fig. 3*D*). This analysis thus suggests that single‐cell level measurements can be biased by the properties of the recorded signals. This leads, for example, to an apparent smearing of offset response distributions in calcium imaging data, which is probably a result of the larger signal variability with this recording modality as measured in Fig. 1*C* and (Bagur et al., 2025). Beyond these discrepancies, this analysis highlights no clear progression of the offset distribution among neurons, except that IC appears to be the structure with the largest fraction of neurons displaying many offset responses compared to CN and TH (Fig. 3*D*).

We then reasoned that different auditory system stages could have different degrees of offset specificity with respect to onset and sustained responses. In Fig. 3*E*, we therefore plotted the fractions of all possible combinations of an offset response (either positive, OFF E, or negative, OFF I) with onset (ON E, ON I or none) and sustained (E, I or none) responses. Note that this analysis is done response‐by‐response and not neuron‐by‐neuron, and that the fractions are given with respect to the total number of responses with a significant offset response. We observed a fraction of ∼40–50% pure positive offset responses (OFF E) in the two‐photon imaging datasets (Fig. 3*E*), whereas, in the electrophysiology datasets, this fraction was down to 20–40%, with variable results across datasets (e.g. IC ephys‐dataset 1: 34.58% and dataset 2: 16.97%) (Fig. 3*E*). Consistent with our sample data plots of Fig. 2*B* and *C*, the fraction of pure negative offset responses was non‐negligible (∼10–25% across datasets). The most prominent responses combined with excitatory offsets were sustained excitatory or inhibitory responses (Sustained E OFF E or Sustained I OFF E) (Fig. 3*E*), or excitatory responses in both the onset and sustained phases (ON E Sustained E OFF E) (Fig. 3*E*) or inhibitory responses in both the onset and sustained phases (ON I Sustained I OFF E) (Fig. 3*E*). However, inhibitory responses before the offset were only detected in electrophysiology datasets (Fig. 3*E*). The most prominent responses combined with inhibitory offsets are monophasic onset and sustained responses, either inhibitory or excitatory (ON I Sustained I OFF E, ON E Sustained E OFF I) (Fig. 3*E*). However, these combinations were also only detected in electrophysiology data, and yet again with some variability, across the IC replicate which could be the result of differences in stimulus properties, recording conditions or neural population sampling (Fig. 3*E*). Altogether, this analysis of combined response type distributions highlighted clear biases across recording modalities probably related to the higher variability of calcium responses, and to their lesser sensitivity to inhibitory responses (but note that many inhibitory offset responses were detected with calcium imaging). Beyond this issue, the analysis shows that, across the auditory system, offsets are often combined with earlier responses. Moreover, at all stages, there was a large fraction of responses with an excitatory offset only. Hence, we did not observe any clear evolution of neurons with offset‐specific responses and no clear evolution of their combination with onset and sustained response, although fine differences may have been missed because of the unreliability of response detection. Because of the rich and distributed nature of offset responses and the difficulties encountered with signal variability at the single‐cell level, we reasoned that a population‐level analysis would be better suited to identify changes in the neural code of offset responses.

### Populations of offset responses are more informative in the AC

We therefore decided to measure the amount of sound information carried by the entire neural population of each auditory system stage sampled in our dataset. We constructed virtual population vectors by concatenating responses measured in all neurons across all recordings (Fig. 4*A*). Population vectors were then fed to a five‐fold, cross‐validated logistic regression decoder to evaluate the fraction of correct sound identity predictions that can be obtained from single‐trial activity of the neural population. We first established a ceiling of the information available in the full spatio‐temporal activity pattern. We sampled the full time‐course of the population into 65 time bins of 10 ms in duration, concatenated the 65 vectors and submitted them to the same decoder. This showed similar information levels across stages, as shown previously, for dataset 2 (Bagur et al., 2025) and lower information level in cortex in dataset 1 (Fig. 4*B*), probably because of the poorer SNR in calcium imaging for dataset 1 (Fig. 1*C*). We then ran population decoders based on the time‐averaged vectors obtained separately for onset, sustained and offset time windows using the previously defined windows for each auditory system stage. To equalize across different dataset qualities, we reported the decoder accuracy computed for a specific time‐bin divided by the ceiling accuracies shown in Fig. 4*B*. This analysis showed that all stages had a similar level of accuracy, with slight variations, in the onset and sustained phases of the response (Fig. 4*C*) but that accuracy was at least three‐fold larger in the AC than in subcortical areas in the offset phase (dataset 1, CN ephys = 13.27% ± 1.84%, ICc ephys = 11.20% ± 2.99%, AC 2*P* = 39.60% ± 2.67%, dataset 2, ICd 2*P* = 29.55% ± 2.21%, ICc ephys = 28.85% ± 3.99%, TH ephys = 26.91% ± 2.24%, AC = 82.29% ± 0.63%, *P* < 0.05 across all pairwise comparisons with cortex, Mann–Whitney *U* test, *n* = five cross‐validation folds) (Fig. 4*C*).

**Figure 4:**
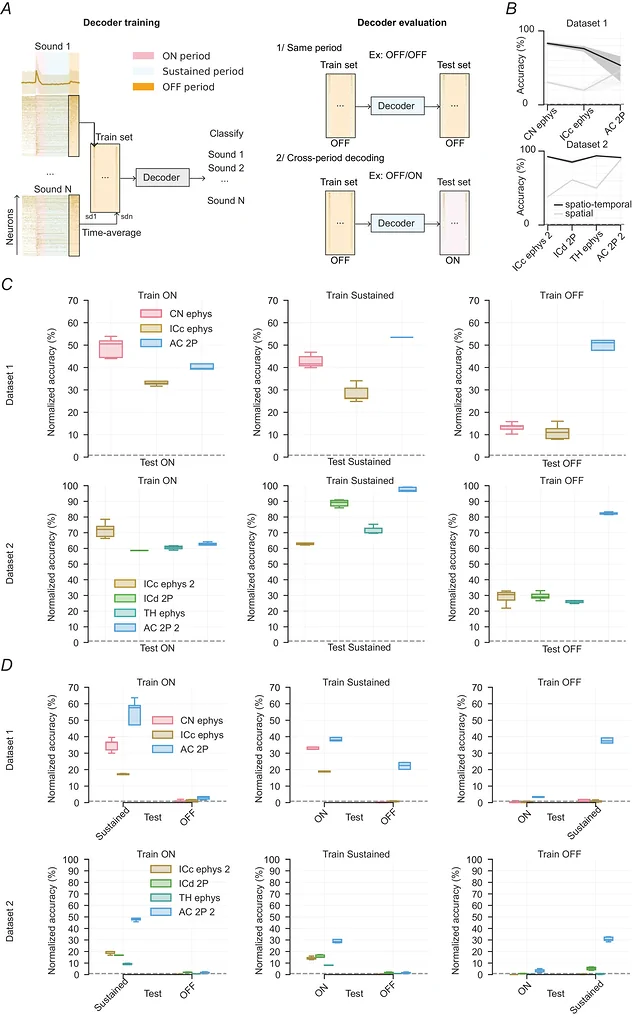
Offset responses are more informative in the auditory cortex *A*, schematic of the decoders used to predict sound identity based on neural activity. *B*, accuracy of population activity decoders using the full spatio‐temporal activity patterns (black) or only the time‐averaged spatial pattern (grey). The accuracy of spatio‐temporal decoders is used in (*C*) and (*D*) as the information ceiling with which the time‐restricted decoder accuracy is normalized. The grey dotted line indicates chance level (1/N sounds). *C*, normalized accuracy of population decoders based on time‐averaged firing rates computed during the onset, sustained and offset phases of the responses respectively for all datasets and recorded brain regions. The normalized accuracy represents the fraction of the ceiling accuracy estimated with the full spatio‐temporal decoder; see (*B*). The grey dotted line indicates chance level. *D*, normalized accuracies for decoders trained in one response phase but tested on another response phase. The grey dotted line indicates chance level.

In both datasets 1 and 2, cortical data are derived only from calcium imaging, raising the possibility that the large difference in decoder performance could be the result of spurious effects of this recording modality. Specifically, temporally deconvolved calcium imaging data are still temporally smeared by the slow rise time of GCAMP6s, which is visible, for example, in the delayed peak of offset responses (Fig. 2*A*). We have compensated for this effect by delaying the offset analysis window for calcium data (Fig. 3). Nevertheless, it is difficult to rule out *a priori* a transfer of sustained response information into the offset window in calcium imaging data as a result of this temporal smearing. Three observations allow us to exclude this possibility. First, decoder accuracy was poor for IC offsets, both for electrophysiology and calcium imaging data (Fig. 4*C*). Second, decoders trained on AC sustained responses had poor accuracy when tested on cortical offset responses (AC 2P 1: 22.21% ± 1.82%, AC 2P 2: 1.47% ± 0.54%) (Fig. 4*D*), suggesting that the information used by the decoder during the offset does not simply mirror sustained activity. However, decoders of offset responses displayed an intermediate accuracy level when tested on sustained activity (AC 2P 1: 33.68% ± 2.30%, AC 2P 2: 30.71% ± 1.67%) (Fig. 4*D*), suggesting a degree of overlap between sustained and offset responses. This can have multiple causes, including temporal smearing, but also a partially shared representation space between sustained and offset phases in AC.

Therefore, our third observation aimed at discriminating between these two alternatives. We decomposed the calcium response on each trial into a linear sum of a set of onset, sustained and offset regressors that modelled the temporal smear based on our data (Fig. 5*A*). Most importantly, we modelled the sustained response with a decaying component that extended into the offset time window (Fig. 5*A*). The regressors have a fixed time course but three time‐dilated regressors were used at the offset to capture the variability of response time constants across neurons (Fig. 5*A*). This approach allowed us to extract one amplitude value for onset, sustained and offset responses at the same time as largely cancelling the smearing effect. We verified the latter aspect by plotting the amplitude of sustained components against offset components and comparing this with the relation of time‐averaged values during sustained and offset periods for calcium imaging data. The clear drop in the correlation between offset and sustained responses after applying the linear demixing model confirmed its efficiency to cancel the temporal smearing effect in calcium signals (Fig. 5*B*). We then measured the accuracy of the decoders trained on the offset component and tested them on the sustained component of the demixing model. We observed overall only a modest drop in classification accuracy compared to response averaging, indicating that the smearing effect, largely cancelled by demixing, contributes little to the information contained in offset responses in the AC dataset (Fig. 5*C*). Likewise, the cross‐decoding of sustained activity by offset decoders was scarcely impacted by the demixing model, showing that temporal smearing does not explain this effect, which instead corresponds to a genuine overlap between sustained and offset representation spaces in the cortex (Fig. 5*D*).

**Figure 5:**
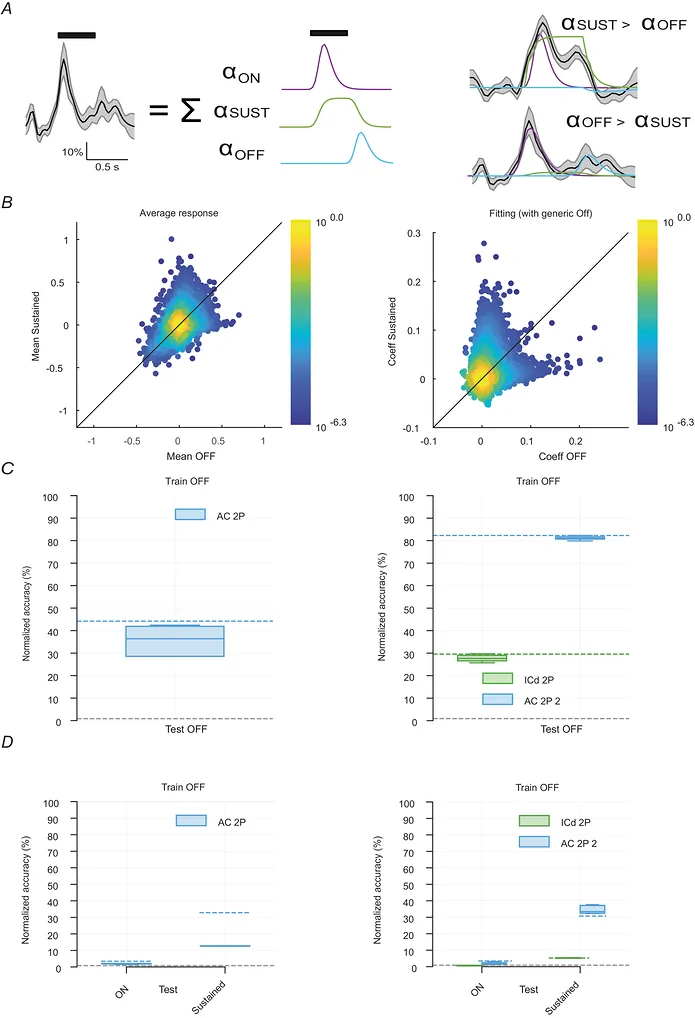
Offset information is specific to offset responses *A*, schematic of the response demixing algorithm used to separate smeared sustained responses from real offset responses in two‐photon calcium imaging. *B*, left: scatter plot of all the time‐averaged deconvolved calcium signals in the offset and sustained time window showing a strong correlation between the two values; right: scatter plot of all the fitted offset and sustained coefficients for the demixing models in the offset and sustained time window, showing a decorrelation of the offset and sustained component of sound responses. *C*, decoder accuracies based only on the offset component of the response model compared to accuracies based on raw data (blue and green dotted lines identical to Fig. 4). The grey dotted line indicates chance level. *D*, accuracy of the decoder based on the offset component of the response model when tested on the onset and sustained components of the response model. The grey dotted line indicates chance level.

## Discussion

Our results provide a systematic analysis of offset responses across the auditory system based on two large datasets collected in awake mice. They complement a wide range of studies that have reported the presence of offset responses throughout the auditory system and characterized their properties across auditory system stages and animal species (Kopp‐Scheinpflug et al., 2018). The main specificities of our dataset are the large number of neurons recorded, especially with calcium imaging, and the homogeneity of the stimulus set and recording conditions across the different stages. Also, some recordings (e.g. IC or AC present in datasets 1 and 2, calcium and electrophysiology in IC) provide repeats of similar experiments, allowing to verify the stability of observed properties across recording modalities, experimental conditions and sampling biases. Our datasets represent a unique collection of mouse auditory system data, but are incomplete to some degree with the absence of recordings in the superior olivary complex and the lack of thalamic data in dataset 1 or of CN in dataset 2.

Reassuringly, our broad dataset recapitulates the main observations made in the past about offset responses. First, offset responses are found at all stages (Fig. 3) in a small subset of neurons. Although consistent with observations that offset responses are sparse, our measurement of the fraction of neurons with offset responses may underestimate the real value because of the poor statistical power of our datasets for this type of question (low sound repeat number and large number of sounds). Previous studies with more statistical power indeed reported larger fractions of offset‐responsive neurons in the IC (Akimov et al., 2017) or in the AC (Tian et al., 2013). In the CN, offset responses are assumed to be present only in specific subtypes of neurons (Ding et al., 1999; Young & Brownell, 1976). It is possible that the large proportion of offset neurons in our CN dataset is the result of a sampling bias introduced by Neuropixels recordings.

Second, offset responses are often, although not systematically, combined with onset and sustained responses (Fig. 3), a property commonly reported at different stages of the auditory system (Kopp‐Scheinpflug et al., 2018). Interestingly, the frequency tuning of on‐ and offset responses in the same neurons of the AC is often different (Scholl et al., 2010; Sollini et al., 2018), suggesting that on‐ and offset responses in the cortex come from circuit processes that are more complex than a single, shared input channel.

Our population decoding analysis demonstrates that cortical offset responses are not only distinct from onset responses, but also they are much more informative about the identity of the elapsed sound than subcortical offsets (Figs 4 and 5). The classifier accuracy improvement between cortical and subcortical levels is more than three‐fold in our two datasets, indicating a robust and clear effect. This striking difference is not a result of the large sample size of AC recordings. Two‐photon calcium imaging data are noisier (Fig. 1*C*) (Bagur et al., 2025) and display sparser offset responses than electrophysiology data (Fig. 3*D*). These two biases are compensated by the larger sample size of the two‐photon calcium imaging data. This is shown in our ceiling information analysis (Fig. 4*B*), which uses population activity decoders based on the full spatio‐temporal information available in each neuron to estimate the maximum sound information available in the dataset. This analysis shows that ceiling information is similar across datasets (Fig. 4*B*). Moreover, decoder accuracies are more similar across auditory system stages for onset and sustained responses (Fig. 4*B*). These two observations demonstrate that the larger sample size of two‐photon calcium imaging does not provide additional information which could explain the highly informative offset in AC. Also, if the sample size of two‐photon calcium imaging data was a factor, offset responses should also be more informative in the two‐photon calcium imaging dataset than in the electrophysiology dataset recorded in the IC, which we do not observe (Fig. 4*C*). The boost of offset information is also not the result of a spillover of sustained information in cortical recordings as demonstrated with our linear demixing analysis (Fig. 5). Hence, we conclude that AC response dynamics are such that they generate highly informative offset activity that does not exist upstream.

This observation is a clear indication that offset responses do not result from a combination of labelled‐line offset inputs from upstream areas. Indeed, if this were the case, as information cannot be created *de novo*, the decoder accuracy would be as poor in the cortex as subcortically. Our results therefore show that offset responses in the AC are recomputed not only from upstream offsets, but also necessarily using information originating in earlier phases of the response, which are much more informative than offset responses at subcortical levels. A previous study reported an absence of offset responses to white noise in the thalamus but not in the AC (Solyga & Barkat, 2021), while offset responses were present in both stages for pure tones. This observation was suggested as potential proof that cortical offset includes new information as compared to subcortical offsets. Our data do not corroborate this observation, as we observe clear offset responses to white noise even in the IC (Fig. 2*C*). Our observations therefore provide a stronger argument for the idea of newly computed offset responses in the AC.

What processes could explain our observation of richer offset responses in the AC? Improved decoder performance in the cortex could result from an improvement of sound‐specific signals, a decrease of offset response variability, or both. Because response variability was larger in cortical datasets, decoder performance was probably improved by an increased sound‐specificity of cortical offsets compared to their subcortical counterparts. The mechanisms usually proposed for bottom‐up generation of offset responses are split between rebound mechanisms in single neurons and local microcircuits mechanisms based, for example, on time‐shifted inhibitory and excitatory inputs (Kopp‐Scheinpflug et al., 2018). Because of their spatial locality, these mechanisms are not sufficient to identify the factors underlying the richness of offset responses in complex circuits with multiple inputs. Recently, it was demonstrated that large recurrent neural networks produce complex offset responses that depend on multiple interactions within the network (Bondanelli et al., 2021). Anatomically, all auditory system networks considered in this study have a recurrent inhibitory and excitatory architecture (Levy & Reyes, 2012; Oliver, 2005; Young & Davis, 2002). It is therefore conceivable that offset responses originate from recurrent network‐scale interactions. This hypothesis would generalize local microcircuit models. This would be compatible with the high sensitivity of offset responses to anaesthetics that strongly affect network dynamics (Filipchuk et al., 2022; Gosselin et al., 2024). In this framework, our results indicate that the collective dynamics generating offset responses are more complex in the AC than in upstream stages. This complexity could span both the spatial dimension (i.e. the extent to which interactions involve multiple neurons processing multiple sound features) and the temporal dimension (i.e. the temporal span over which information is integrated to produce the offset response). It is already well established that AC responses have less temporal precision than subcortical responses (Wang et al., 2008) and are influenced by a longer history time window (Norman‐Haignere et al., 2022). This could contribute to the increased richness of cortical offset responses, which, because of their long duration (Kopp‐Scheinpflug et al., 2018) (Fig. 2), also provide, at the population scale, a transient memory of the elapsed sound.

## Additional information

## Competing interests

The authors declare that they have no competing interests.

## Author contributions

C.L., E.G., B.B. and S.B. conceptualized, developed the methodology, and reviewed and edited the manuscript. C.L., E.G. and S.B. investigated, curated the data and performed the formal analysis. B.B. and S.B. supervised the work, acquired the funding and validated the results. B.B. administered the project. C.L. and S.B. developed the software. C.L. prepared the visualizations. Reviewing and editing C.L., S.B., B.B., E.G. All authors have approved the final version of the manuscript submitted for publication and agree to be accountable for all aspects of the work. All persons designated as authors qualify for authorship, and all those who qualify for authorship are listed.

## Funding

This study was supported by European Research Council (ERC), Fondation pour la Recherche Médicale (FRM), Fondation Pour l'Audition (FPA) and Agence Nationale pour la Recherche, France 2030 program.

## Supporting information


Peer Review History

## Data Availability

All data supporting the results in the paper are archived in the public repository Zenodo (access links: https://doi.org/10.5281/zenodo.13941450 and https://doi.org/10.5281/zenodo.14421103).
