# Supplementary material for: Sound offset responses become highly informative in the auditory cortex
Source: J Physiol. 2025 Sep 24;604(21):8702–20. doi: 10.1113/JP288416 (PMC13725235; doi:10.1113/JP288416)
Supplement: Supplementary file 1 [file TJP-604-8702-s001.pdf]

The Journal of Physiology

Sound offset responses become highly informative in the auditory cortex

Charly Lamothe, Sophie Bagur, Etienne Gosselin, and Brice Bathellier
DOI: 10.1113/JP288416

Corresponding author(s): Brice Bathellier (Brice.bathellier@cnrs.fr)

The referees have opted to remain anonymous.

Review Timeline:

Submission Date:	25-Dec-2024
Editorial Decision:	12-Mar-2025
Revision Received:	12-May-2025
Editorial Decision:	24-Jul-2025
Revision Received:	02-Aug-2025
Accepted:	18-Aug-2025

Senior Editor: Vaughan Macefield

Reviewing Editor: Conny Kopp-Scheinpflug

Transaction Report:

(Note: With the exception of the correction of typographical or spelling errors that could be a source of ambiguity, letters and reports are not edited. Depending on transfer agreements, referee reports obtained elsewhere may or may not be included in this compilation. Referee reports are anonymous unless the Referee chooses to sign their reports.)

Dear Dr Bathellier,

Re: JP-RP-2024-288416 "Sound offset responses become highly informative in the auditory cortex" by Charly Lamothe, Sophie Bagur, Etienne Gosselin, and Brice Bathellier

Thank you for submitting your manuscript to The Journal of Physiology. It has been assessed by a Reviewing Editor and by 2 expert referees and we are pleased to tell you that it is potentially acceptable for publication following satisfactory major revision.

Please advise your co-authors of this decision as soon as possible.

The referee reports are copied at the end of this email.

Please address all the points raised and incorporate all requested revisions or explain in your Response to Referees why a change has not been made. We hope you will find the comments helpful and that you will be able to return your revised manuscript within 2 months. If you require longer than this, please contact journal staff: jp@physoc.org. Please note that this letter does not constitute a guarantee for acceptance of your revised manuscript.

Your revised manuscript should be submitted online using the link in your Author Tasks: Link Not Available. This link is accessible via your account as Corresponding Author; it is not available to your co-authors. If this presents a problem, please contact journal staff (jp@physoc.org). Image files from the previous version are retained on the system. Please ensure you replace or remove any files that are being revised.

If you do not wish to submit a revised version of your manuscript, you must inform our journal staff (jp@physoc.org) or reply to this email to request withdrawal. Please note that a manuscript must be formally withdrawn from the peer review process at one journal before it may be submitted to another journal.

TRANSPARENT PEER REVIEW POLICY: To improve the transparency of its peer review process, The Journal of Physiology publishes online as supporting information the peer review history of all articles accepted for publication. Readers will have access to decision letters, including Editors' comments and referee reports, for each version of the manuscript, as well as any author responses to peer review comments. Referees can decide whether or not they wish to be named on the peer review history document.

ABSTRACT FIGURES: Authors are expected to use The Journal's premium BioRender account to create/redraw their Abstract Figures. Information on how to access this account is here: https://physoc.onlinelibrary.wiley.com/journal/14697793/biorender-access.

This will enable Authors to create and download high-resolution figures. If authors have used the free BioRender service, they can use the instructions provided in the link above to download a high-resolution version suitable for publication.

The link provided should only be used for the purposes of this submission. Authors will be charged for figures created on this account if they are not related to this manuscript submission.

LANGUAGE EDITING AND SUPPORT FOR PUBLICATION: If you would like help with English language editing, or other article preparation support, Wiley Editing Services offers expert help, including English Language Editing, as well as translation, manuscript formatting, and figure formatting at www.wileyauthors.com/eeo/preparation. You can also find resources for Preparing Your Article for general guidance about writing and preparing your manuscript at www.wileyauthors.com/eeo/prepresources.

RIGOUR AND REPRODUCIBILITY: We recommend authors consult The Journal's Rigour and Reproducibility requirements to ensure that their manuscript is suitable for publication: https://physoc.onlinelibrary.wiley.com/pb-assets/hub-assets/physoc/documents/TJP-Rigour-and-Reproducibility-Requirements.pdf

REVISION CHECKLIST:

Check that your Methods section conforms to journal policy: https://jp.msubmit.net/cgi-bin/main.plex?form_type=display_requirements#methods.

Check that data presented conforms to the statistics policy: https://jp.msubmit.net/cgi-bin/main.plex?form_type=display_requirements#statistics.

Upload a full Response to Referees file. To create your 'Response to Referees': copy all the reports, including any comments from the Senior and Reviewing Editors, into a Microsoft Word, or similar, file and respond to each point, using font or background colour to distinguish comments and responses and upload as the required file type.

Please upload two versions of your manuscript text: one with all relevant changes highlighted and one clean version with no

changes tracked. The manuscript file should include all tables and figure legends, but each figure/graph should be uploaded as separate, high-resolution files.

You may also upload:

- 'Potential Cover Art' for consideration as the issue's cover image
- Appropriate Supporting Information (Video, audio or data set: see https://jp.msubmit.net/cgi-bin/main.plex?form_type=display_requirements#supp).

We look forward to receiving your revised submission.

If you have any queries, please reply to this email and we will be pleased to advise.

Yours sincerely,

Vaughan Macefield
Senior Editor
The Journal of Physiology

REQUIRED ITEMS

- Include a Key Points list in the article itself, before the Abstract.

- Author photo and profile. First or joint first authors are asked to provide a short biography (no more than 100 words for one author or 150 words in total for joint first authors) and a portrait photograph. These should be uploaded and clearly labelled together in a Word document with the revised version of the manuscript. See Information for Authors for further details.

- You must start the Methods section with a paragraph headed Ethical approval (https://jp.msubmit.net/cgi-bin/main.plex?form_type=display_requirements#methods).

Research must comply with The Journal's policies regarding animal experiments (https://physoc.onlinelibrary.wiley.com/hub/animal-experiments) and adherence to these policies must be stated in the manuscript.

Authors should confirm in their Methods section that their experiments were carried out according to the guidelines laid down by their institution's animal welfare committee, including an ethics approval reference number. The Methods section must contain a statement about access to food, water and housing, details of the anaesthetic regime: anaesthetic used, dose and route of administration, and method of killing the experimental animals.

- Your manuscript must include a complete Additional Information section, including competing interests; funding; author contributions and acknowledgements.

- Please upload separate high-quality figure files via the submission form.

- Please ensure that any tables are editable and in Word format, and wherever possible, embedded in the article file itself.

- Please ensure that the Article File you upload is a Word file.

- A Data Availability Statement is required for all papers reporting original data. This must be in the Additional Information section of the manuscript itself. It must have the paragraph heading 'Data Availability Statement'. All data supporting the results in the paper must be either: in the paper itself; uploaded as Supporting Information for Online Publication; or archived

in an appropriate public repository. The statement needs to describe the availability or the absence of shared data. Authors must include in their statement: a link to the repository they have used, or a statement that it is available as Supporting Information; reference the data in the appropriate sections(s) of their manuscript; and cite the data they have shared in the References section. Whenever possible, the scripts and other artefacts used to generate the analyses presented in the paper should also be publicly archived. If sharing data compromises ethical standards or legal requirements then authors are not expected to share it, but must note this in their statement. For more information, see our Statistics Policy.

- Please include an Abstract Figure file, as well as the Figure Legend text within the main article file. The Abstract Figure is a piece of artwork designed to give readers an immediate understanding of the research and should summarise the main conclusions. If possible, the image should be easily 'readable' from left to right or top to bottom. It should show the physiological relevance of the manuscript so readers can assess the importance and content of its findings. Abstract Figures should not merely recapitulate other figures in the manuscript. Please try to keep the diagram as simple as possible and without superfluous information that may distract from the main conclusion(s). Abstract Figures must be provided by authors no later than the revised manuscript stage and should be uploaded as a separate file during online submission labelled as File Type 'Abstract Figure'. Please also ensure that you include the figure legend in the main article file. All Abstract Figures should be created using BioRender. Authors should use The Journal's premium BioRender account to export high-resolution images. Details on how to use and access the premium account are included as part of this email.

EDITOR COMMENTS

Reviewing Editor:

Methods Details:
Please see comments of reviewer 1. The authors should use a figure to clarify which sounds (or examples) were used and how neurons were classified into being an offset neurons or not. This can be part of the methods or (as I would prefer) part of the results.

Comments to the Author (Required):
Your manuscript has been reviewed by two experts in the field and my co-editor and I have discussed the reviews and your manuscript. The reason why the decision took so long was that we really agree with reviewer 1 in that you need to re-work the statistics and clarify your rationale of when a neuron was classified as offset. So far it seems that neurons which respond to one out of 119 sound with an offset are classified as offset... maybe use a figure to clearly explain the classification process?

The dataset and work you are describing is potentially a very valuable addition to this special issue, however, as the reviewers point out - the take-home message of the current manuscript may very well change into another direction after re-working the statistics.

So, please carefully revise the manuscript and we will be looking forward to your resubmission.

Please also see 'Required Items' above.

Kind regards

Senior Editor:

Thank you for submitting your manuscript to The Journal of Physiology. I have now received comments from two independent reviewers and the Reviewing Editor, all experts in the field. As you will see from their comments, both reviewers see merit in your work but both raise issues you will need to address before we can consider the manuscript further. In particular, you need to attend the concerns of Reviewer 1 regarding your statistical approach. You will also need to ensure that the source of animals is provided and that terminal procedures are described. I invite you to revise the manuscript accordingly and submit point-by-point responses to the reviewers' comments, and look forward to receiving your revised manuscript in due course.

REFEREE COMMENTS

Referee #1:

This paper by Lamothe et al addresses the questions of how offset responses emerge in the auditory system and what their functions are. The central findings are that offset responses are extremely prevalent (present in nearly all neurons at all stages of the auditory system) and that these offset responses become more informative as one ascends from the cochlear nucleus to the AC. These results are interesting and potentially important, and the unmixing algorithm in figure 5 is an especially nice way to disambiguate and validate the importance of offset responses as opposed to leftover sustained responses. The paper draws on two separate datasets with distinct methodologies, and validates that most results are similar on the two datasets. This is also a meaningful strength.

That said, the paper has what I believe to be serious issues with the data analysis that have led the authors to incorrect conclusions about the prevalence of offset responses, and these lead to serious issues with the work as written. These errors could be overcome with reanalysis, and I hope that the authors do because the significance of the work could otherwise be strong. The re-analyzed conclusions may not be very similar to the current conclusions.

The key statistical error is that no multiple comparisons corrections (MCC) or false discovery rate (FDR) corrections were performed, although each neuron was tested with more than a hundred different sounds, and was classed as having offset responses if even *one* of those sound responses met significance criteria (which were complex but not stringent - p values of <=0.05). Under these conditions, one would expect to frequently observe apparently statistically significant results by chance. Indeed, 92 to 100% of neurons, depending on the area, were classified as possessing offset responses under these criteria - far more than what other groups have reported, and far in excess of what one would estimate from their figures (such as 2B, 2C). This is a serious error in data interpretation and obviously compromises their first key result, that the prevalence of offset responses is very high and similar at different stages of the auditory system. This error also may explain the specific discrepancy with prior work in which they report nearly all CN neurons having offset responses while other authors have reported CN offset responses being rare.

This statistical error also affects all claims about the frequency with which the same neuron shows onset, sustained, and/or offset responses. These analyses should be redone.

This same issue also affects the main decoding results. The result that responses during the sound offset period become more useful in decoding as one ascends in the auditory system could result either from offset responses becoming more prevalent/frequent, or from offset responses becoming more informative. The lack of multiple comparisons correction means that many of the neurons classified as having OFF responses (and thus included in the decoder) might not actually have offset responses, and thus that the changes in decoding quality could be majorly confounded by the changes in prevalence in offset responses as one progresses from CN to AC. To match the way the results are described in the text, these results would need to be re-calculated using only neurons meeting a much more stringent definition of OFF responses. There is probably still an important result here, but is the result "Offset responses become more common later in the auditory system, but when they do occur, are equally informative at all stages" vs "Offset responses become both more common and more specific/informative later in the auditory system"?

Along those lines, it would have been very interesting and perhaps reassuring to see how similar each neuron's ON/Sust/Off classification was when probed with different sounds in the sound set. IE, if a neuron has a large, significant excitatory On and Off responses to one sound, will it tend to show the same On-Off pattern to all the sounds it responds to? If a neuron's temporal response patterns are consistent across stimuli, it's less likely that it has been misclassified based on its "statistically significant" response to a single stimulus. Alternatively, if it's really the case that some neurons have Off responses to only a few stimuli - this would be very surprising but it might be true! - then it would be essential to see that point persuasively made with more expansive figures, example cells, and MCC/FDR corrected statistics.

In a dataset with as many neurons as these, one might also improve rigor by performing neuron-by-neuron FDR correction.

The exact methods for picking out ON and OFF responses are a little bit unusual (combinations of several criteria). To persuade the reader that the right analysis choices were made, it could be helpful to have figures illustrating the groups of neurons that do and do not match the different criteria.

The paper contains *many* typographical errors, incorrect word orders, etc. EG on page 3 alone, errors include "used in a to describe", "simple a linear algorithm", "according to offset the amplitudes", "offsets responses". It is usually possible to infer what is meant, but these errors make the paper a difficult read and it should be carefully proofread before resubmission.

The figures could generally be improved (bolder lines, larger fonts).

The paper is an analysis of two datasets. One of these datasets has been published and is appropriately cited here, but the other dataset is listed as belonging to a paper "in preparation" and no citation or reference to a preprint is given. If this is the first analysis of a previously published dataset then the prior publication must be cited; otherwise *this* is the first publication of the dataset.

Referee #2:

This study focuses on offset responses within the auditory system-responses that have historically received insufficient attention and whose roles in sound processing remain unclear. The research thoroughly describes the presence of offset responses throughout the auditory system, utilizing two extensive datasets that combine electrophysiology and calcium imaging in awake mice. This meticulously designed and executed study draws on large datasets recorded via either electrophysiology or two-photon imaging. The key finding of this research is that offset responses are not merely signals indicating the cessation of sound; instead, they carry significant information regarding the identity of the stimulus in the auditory cortex, but not in sub-cortical auditory regions. This outcome challenges the simplistic view of offset responses as mere termination signals and suggests that cortical computations enrich the representation of sound at its offset.

Overall, the paper is well-written, featuring clear explanations and robust methodologies, and it importantly contributes to the field. However, there are areas where the study could be strengthened by addressing potential confounds especially in the decoding analysis and offering a more comprehensive discussion of its limitations. My comments below are mainly around specific points that could be addressed hopefully with minor work.

1. The decoding results represent the key evidence of this study, highlighting the significant findings related to the offset responses in the auditory cortex (AC) compared to subcortical regions. However, the comparison between electrophysiological (ephys) signals and two-photon (2P) imaging may introduce a potential confound. I commend the authors for acknowledging this limitation within the manuscript and implementing careful controls to mitigate the issue (such as deconvolution for calcium signals, normalized by ceiling accuracy, etc.). If collecting new electrophysiological data from the AC to facilitate a direct comparison with other brain regions is not feasible within the revision timeline, I suggest that the authors: 1) should discuss how differences in signal-to-noise ratio (SNR) between ephys and calcium imaging might influence the decoding results. For example, if the SNR is lower in calcium imaging data, this could reduce decoding accuracy independently of neural processing differences. 2) Additionally, they should consider whether the larger number of neurons recorded in the cortex might contribute to the higher decoding accuracy, and they could test this by subsampling the cortical data to match the number of units in subcortical regions.

2. Dataset Differences and Variability:The manuscript highlights differences between Dataset 1 and Dataset 2, particularly in the prevalence of combined ON E, sustained E, and OFF E responses and the distribution of offset response fractions (Fig. 3). While the authors attribute these differences to higher variability in Dataset 1, they do not explicitly quantify or visualize this variability. For example:1.The broader distribution of offset response fractions in Dataset 1 cortex (Fig. 3D) suggests higher variability, but the authors should clarify how this variability was measured (e.g., inter-trial correlations, response consistency).2.Differences in stimulus properties, recording conditions, or neural population sampling between the two datasets could also contribute to the observed differences. The authors should discuss these factors in more detail.

3. Fig 3C. Discrepancy in Negative Offset Responses: This is an interesting observation, where the authors provide a reasonable explanation for the discrepancy in negative offset responses (OFF I) between calcium imaging and electrophysiology data, linking it to differences in recording modalities. However, they could strengthen this explanation by: 1. Providing quantitative evidence of the differences in variability between calcium imaging and electrophysiology.2. Discussing whether the discrepancy might also reflect genuine differences in neural processing (e.g., more inhibitory activity

in cortical circuits compared to subcortical regions).

4. The quality and clarity of the figures are generally good, but there is room for enhancement to improve their visual presentation. For instance, the contours in Figure 3C are nearly invisible. In Figure 2, the heatmaps would benefit from clearer labels and a more detailed legend to assist readers in interpreting the data. Additionally, adjusting the c-axis would better highlight the evoked and suppressed responses. In Figure 3D, is the y-axis label correct? Should it not be the proportional of neurons instead of proportion of stimuli?

5. Terminology: The authors used positive/negative response terms, which are less used, and instead, 'excited' (E) and 'inhibited' (I) are more intuitive and align with the legends in the figures. Using these terms consistently throughout the manuscript would improve clarity and make it easier for readers to interpret the results.

6. The introduction lacks more comprehensive review about offset responses and their importance in gap detection (DOI: 10.1093/cercor/bhaa044), perceptual grouping(doi: https://doi.org/10.7551/mitpress/1486.001.0001), noise rejection (doi:10.1007/BF00696152), and their role in prediction-error signaling (omission responses, https://doi.org/10.1016/j.cub.2023.06.013). This would provide a broader context for the study and highlight the importance of offset responses in auditory processing. Additionally, the authors could discuss how their findings relate to the observation that omission responses are primarily cortical (doi:10.1016/j.neuroscience.2016.09.011), suggesting a potential link between offset responses and predictive coding.

END OF COMMENTS

Reviewing Editor:

Methods Details:

Please see comments of reviewer 1. The authors should use a figure to clarify which sounds (or examples) were used and how neurons were classified into being an offset neurons or not. This can be part of the methods or (as I would prefer) part of the results.

We have simplified and cleaned the procedure to detect offset responses for a given sound, which is the base of our statistical analysis in Figure 3 to estimate the fraction of responses with offset and the fraction of neurons concerned. This procedure is described in Fig. 3A. Moreover we have used a correction for multiple testing in the procedure that determines the fraction of neurons with at least one offset response. These two modifications have drastically changed one aspect of the single-cell results: not all neurons have statistically significant offset responses, just a small fraction. The statistical procedures are now detailed in both the results and the methods.

Comments to the Author (Required):

Your manuscript has been reviewed by two experts in the field and my co-editor and I have discussed the reviews and your manuscript. The reason why the decision took so long was that we really agree with reviewer 1 in that you need to re-work the statistics and clarify your rationale of when a neuron was classified as offset. So far it seems that neurons which respond to one out of 119 sounds with an offset are classified as offset... maybe use a figure to clearly explain the classification process?

The dataset and work you are describing is potentially a very valuable addition to this special issue, however, as the reviewers point out - the take-home message of the current manuscript may very well change into another direction after re-working the statistics.

The main conclusion of our manuscript is not dependent on the classification of offset neurons because the sound identity classifiers are using information from all neurons. However, we fully agree with the editors and the referees that our previous definition of offset responses and neurons as reported in Fig. 3 was somewhat unclear and too permissive, yielding conclusions that were hard to reconcile with previous observations. We have reworked this part of the manuscript, using a clearly defined statistical test that yields a much smaller fraction of offset neurons.

Senior Editor:

Thank you for submitting your manuscript to The Journal of Physiology. I have now received comments from two independent reviewers and the Reviewing Editor, all experts in the field. As you will see from their comments, both reviewers see merit in your work but both raise issues you will need to address before we can consider the manuscript further. In particular, you need to attend the concerns of Reviewer 1 regarding your statistical approach. You will also need to ensure that the source of animals is provided and that terminal procedures are described. I invite you to revise the manuscript accordingly and submit point-by-point responses to the reviewers' comments, and look forward to receiving your revised manuscript in due course.

We have addressed point-by-point the referee's comments, which have largely improved the manuscript and have provided the source of animals and terminal procedures in the methods.

All modifications of the manuscript are highlighted in red. Some are cited directly in this rebuttal but not all.

REFEREE COMMENTS

Referee #1:

This paper by Lamothe et al addresses the questions of how offset responses emerge in the auditory system and what their functions are. The central findings are that offset responses are extremely prevalent (present in nearly all neurons at all stages of the auditory system) and that these offset responses become more informative as one ascends from the cochlear nucleus to the AC. These results are interesting and potentially important, and the unmixing algorithm in figure 5 is an especially nice way to disambiguate and validate the importance of offset responses as opposed to leftover sustained responses. The paper draws on two separate datasets with distinct methodologies, and validates that most results are similar on the two datasets. This is also a meaningful strength.

That said, the paper has what I believe to be serious issues with the data analysis that have led the authors to incorrect conclusions about the prevalence of offset responses, and these lead to serious issues with the work as written. These errors could be overcome with reanalysis, and I hope that the authors do because the significance of the work could otherwise be strong. The re-analyzed conclusions may not be very similar to the current conclusions.

We thank the reviewer for the assessment of our work. We have addressed the main concern about the statistical procedure with a clearer statistical framework highlighting the expected FDR and correction for multiple testing where it was missing.

1. The key statistical error is that no multiple comparisons corrections (MCC) or false discovery rate (FDR) corrections were performed, although each neuron was tested with more than a hundred different sounds, and was classed as having offset responses if even *one* of those sound responses met significance criteria (which were complex but not stringent - p values of <=0.05). Under these conditions, one would expect to frequently observe apparently statistically significant results by chance. Indeed, 92 to 100% of neurons, depending on the area, were classified as possessing offset responses under these criteria - far more than what other groups have reported, and far in excess of what one would estimate from their figures (such as 2B, 2C). This is a serious error in data interpretation and obviously compromises their first key result, that the prevalence of offset responses is very high and similar at different stages of the auditory system. This error also may explain the specific discrepancy with prior work in which they report nearly all CN neurons having offset responses while other authors have reported CN offset responses being rare.

We thank the reviewer for raising this important issue. Our identification of offset responses was over-optimistic due to incomplete statistical control (threshold on the response

amplitude based on response variance w/o FDR calculation) and to the use of the absolute value responses instead of the actual, signed values.

To address this issue, we have simplified and reinforced the single neuron response detection process. For onset and sustained response detection, it is now based on one non-parametric statistical test (Wilcoxon signed-rank test, alpha value = 0.05 = FDR) comparing baseline activity to onset or sustained activity respectively. For offset response detection, we used two non-independent signed-rank tests comparing offset activity to baseline and sustained activity respectively. This testing procedure follows the same rationale as the previous one presented but now includes explicit p-value computations for both tests. Both tests require a significant p-value for a response to be classified as "offset". Because the measurements of baseline and sustained responses come from the same experiment, they are not statistically independent. Therefore, the false discovery rate (FDR) is not the product of alpha values as in the case of two independent tests, but is likely higher. To be conservative, we used the alpha value of one signed-rank test (0.05, same for both tests), as an estimate of the false detection rate (FDR) for offsets.

Based on this more precise approach, described in the results, in Fig. 3A and in the methods we report in Fig. 3B the fraction of all neuron-sound pairs for which we observe a significant response at the offset. Our conservative estimation of the FDR is 5% as described above. This means that the fraction of responses reported are for sure above chance when they exceed 5%, which is the case in all datasets. We also report in Fig. 3C the fraction of neurons with at least one offset response. In this case, we applied the Benjamini-Hochberg comparison for multiple testing to compensate for the large number of tests that are performed to determine if a neuron responds once with an offset. Therefore, the FDR of the overall test is also 5%. This approach yields smaller fractions of offset responses (by a factor ~2) and of neurons (due to the absence of multiple testing correction previously) than we reported previously, but still above the 5% chance level except for the auditory cortex in dataset 1.

Similar fractions of offset responses were found in all datasets, in line with our previous observations. However, because of the smaller fractions of offset responses, we removed the conclusion that offset responses are largely distributed across the neuronal population from the abstract results and discussion.

The detection of offset responses (Fig. 3B) is used only in Figure 3E. The detection of neurons with at least one offset response (Fig. 3C) is used only in Figure 3D. Therefore these modifications do not impact Fig. 4 and 5 and do not change the main conclusion of our manuscript, which is that offset responses are more informative in the cortex. Also, please note that, even if we have now carefully harnessed the FDR (and it was necessary), the statistical tests do not handle false negative detection (FND), which depends on data noise and on the number of repeats for each sound. These factors are constraints of the datasets, which overall make the values reported in Figure 3 somewhat relative. This is one of the reasons why we opted afterwards in the manuscript for a population approach that includes all neurons independent of the statistical threshold.

2. This statistical error also affects all claims about the frequency with which the same neuron shows onset, sustained, and/or offset responses. These analyses should be redone.

The analyses were redone and yielded very similar results, except that a larger fraction of offset responses were pure offsets, and fewer neurons had onset and offset responses at the same time without showing any sustained activity (see new Figure 3D and E).

3. This same issue also affects the main decoding results. The result that responses during the sound offset period become more useful in decoding as one ascends in the auditory system could result either from offset responses becoming more prevalent/frequent, or from offset responses becoming more informative. The lack of multiple comparisons correction means that many of the neurons classified as having OFF responses (and thus included in the decoder) might not actually have offset responses, and thus that the changes in decoding quality could be majorly confounded by the changes in prevalence in offset responses as one progresses from CN to AC. To match the way the results are described in the text, these results would need to be re-calculated using only neurons meeting a much more stringent definition of OFF responses. There is probably still an important result here, but is the result "Offset responses become more common later in the auditory system, but when they do occur, are equally informative at all stages" vs "Offset responses become both more common and more specific/informative later in the auditory system"?

First there is a misunderstanding related to this comment. The decoding results reported in Figure 4 are performed on all neurons and not just those with an identified offset response. We have now explicitly stated that in the methods: "To decode the stimulus identity from neural activity, we evaluated performance across different response windows, including onset, sustained, offset, and full response periods. For each window, neural responses from all neurons in each considered dataset were extracted and concatenated into a virtual population vector. In order to avoid statistical threshold effects, the selection of offset responses based on single neuron responses was not used in the population decoding approach."

Therefore, the stringency of the neuron classification has no impact on our classifiers and our main conclusion.

Whether the more informative offset responses in AC are due to a higher prevalence or to a higher specificity as we suggest is an important question, and we agree with the referee that it must be carefully treated. After performing more stringent offset response detection, we still find a similar prevalence of offset responses at all auditory system stages considered. Therefore we do not think that this is the main factor, but rather that offset responses become more specific in the cortex.

4. Along those lines, it would have been very interesting and perhaps reassuring to see how similar each neuron's ON/Sust/Off classification was when probed with different sounds in the sound set. IE, if a neuron has a large, significant excitatory On and Off responses to one sound, will it tend to show the same On-Off pattern to all the sounds it responds to? If a neuron's temporal response patterns are consistent across stimuli, it's less likely that it has been misclassified based on its "statistically significant" response to a single stimulus. Alternatively, if it's really the case that some neurons have Off responses to only a few stimuli - this would be very

surprising but it might be true! - then it would be essential to see that point persuasively made with more expansive figures, example cells, and MCC/FDR corrected statistics.

We have shown multiple examples in Figure 2 (more examples shown now) indicating clearly that when a neuron has a large offset response on one sound it likely shows another nice offset response on other sounds as the reviewer describes. Yet, making the point statistically that this is systematic when we statistically detect an offset response is a difficult challenge that goes much beyond the ambition of this manuscript. The reason is that statistical tests harness FDR but not false negative detections. To perform the requested analysis meaningfully, we would need a much larger number of sound repeats, which is not available in these datasets.

5. In a dataset with as many neurons as these, one might also improve rigor by performing neuron-by-neuron FDR correction.

As detailed above, we have performed a correction for multiple testing (Benjamini-Hochberg) for the detection of neurons with at least one offset response (Fig. 3C lower panels). For the offset response detection analysis (3C upper panel), an estimated chance level is now explicitly given based on the estimated FDR. These modifications have clearly improved the single-cell level analysis reported in Figure 3, although the lack of information about the false negative detection rate sets limits on the generality of the values reported in this Figure. The paragraph describing Figure 3 has been largely rewritten.

6. The exact methods for picking out ON and OFF responses are a little bit unusual (combinations of several criteria). To persuade the reader that the right analysis choices were made, it could be helpful to have figures illustrating the groups of neurons that do and do not match the different criteria.

We have simplified the response detection scheme with only two statistical tests. We have tried to reduce the approach to a single statistical test based on a single index accounting for the difference of offset activity with baseline and sustained activity, but this turned out to be more conservative than the two-test approach due to the high degree of correlation between variability in the baseline and sustained phases. The approach is now much more conventional and should be more convincing.

We nevertheless have increased the number of examples in Fig 2. These examples correspond to neurons that have been detected by our new statistical approach and have clear offset responses across several stimuli.

7. The paper contains *many* typographical errors, incorrect word orders, etc. EG on page 3 alone, errors include "used in a to describe", "simple a linear algorithm", "according to offset the amplitudes", "offsets responses". It is usually possible to infer what is meant, but these errors make the paper a difficult read and it should be carefully proofread before resubmission.

We apologize for these mistakes and have now carefully proofread the manuscript.

8. The figures could generally be improved (bolder lines, larger fonts).

We have polished the figures.

9. The paper is an analysis of two datasets. One of these datasets has been published and is appropriately cited here, but the other dataset is listed as belonging to a paper "in preparation" and no citation or reference to a preprint is given. If this is the first analysis of a previously published dataset then the prior publication must be cited; otherwise *this* is the first publication of the dataset.

We have now published in BioRxiv the paper noted as in preparation. The reference to this preprint is now provided in the manuscript.

Referee #2:

This study focuses on offset responses within the auditory system-responses that have historically received insufficient attention and whose roles in sound processing remain unclear. The research thoroughly describes the presence of offset responses throughout the auditory system, utilizing two extensive datasets that combine electrophysiology and calcium imaging in awake mice. This meticulously designed and executed study draws on large datasets recorded via either electrophysiology or two-photon imaging. The key finding of this research is that offset responses are not merely signals indicating the cessation of sound; instead, they carry significant information regarding the identity of the stimulus in the auditory cortex, but not in sub-cortical auditory regions. This outcome challenges the simplistic view of offset responses as mere termination signals and suggests that cortical computations enrich the representation of sound at its offset.

Overall, the paper is well-written, featuring clear explanations and robust methodologies, and it importantly contributes to the field. However, there are areas where the study could be strengthened by addressing potential confounds especially in the decoding analysis and offering a more comprehensive discussion of its limitations. My comments below are mainly around specific points that could be addressed hopefully with minor work.

We thank the reviewer for the assessment of our work. We have addressed the comments with complementary analyses.

1. The decoding results represent the key evidence of this study, highlighting the significant findings related to the offset responses in the auditory cortex (AC) compared to subcortical regions. However, the comparison between electrophysiological (ephys) signals and two-photon (2P) imaging may introduce a potential confound. I commend the authors for acknowledging this limitation within the manuscript and implementing careful controls to mitigate the issue (such as deconvolution for calcium signals, normalized by ceiling accuracy, etc.). If collecting new electrophysiological data from the AC to facilitate a direct comparison with other brain regions is not feasible within the revision timeline, I suggest that the authors: 1) should discuss how differences in signal-to-noise ratio (SNR) between ephys and calcium imaging might influence the decoding results. For example, if the SNR is lower in calcium imaging data, this could reduce decoding accuracy independently of neural processing differences. 2) Additionally, they should consider whether the larger number of neurons recorded in the cortex might contribute to the higher decoding accuracy, and they could test this by subsampling the cortical data to match the number of units in subcortical regions.

The issue raised by the referee is important, but its full resolution largely goes beyond this manuscript. There is a long standing issue about sampling biases introduced by different

recording techniques. It was raised first by the Zador lab (Hromádka et al. 2008) in the auditory cortex, using loose patch recordings, which showed that extracellular multielectrode electrophysiology is not necessarily a gold standard for this brain region, despite some of its advantages such as relatively high throughput, good temporal resolution, and high SNR. Due to its strong bias towards highly firing neurons some representational properties, specific to the auditory cortex, such as dominance of low firing rates and response sparseness are not properly reproduced by multielectrode recordings (Hromádka et al. 2008). Calcium imaging corroborated this observation (Hromádka et al. 2008). Calcium imaging of course also introduces its own biases, which also must be better characterized, and they must be taken in account, based on current, incomplete knowledge, when interpreting the data, as we did in Bagur et al. 2025 from which one of the dataset is extracted. We reinforced this aspect in the present manuscript based on the reviewer recommendations.

We have provided in Bagur et al. an evaluation of the differences of SNR between electrophysiology (high) and two-photon imaging (lower). We now refer explicitly to this analysis for dataset 2 in the results based on the average correlation between single trial responses in each neuron. We have also computed the same measure for dataset 1 and provided it in Fig. 1C (the graphs are reproduced in the next point). The differences of SNR and of sparseness is such that a much higher number of neurons is required with two-photon calcium imaging to retrieve the same information level. We have shown this in Bagur et al. 2025 (Figure 4C) and we have performed the same analysis in this manuscript in Figure 4B by training classifiers to discriminate sounds based on the full spatio-temporal population responses. These analyses show that the maximum information level retrievable with our decoders is comparable across datasets, except for the smaller auditory cortex dataset (2P dataset 1).

The information present in offset responses is a subset of the information retrieved by full spatio-temporal decoding. Therefore, the differences of dataset size and SNR cannot explain the higher classification performance in the cortex at the offset. It is conceivable though that sampling biases or activity distortions introduced by two-photon calcium imaging could magnify offset information. We have controlled for this by running classifiers on the offset response of the two-photon imaging and electrophysiology datasets recorded in the inferior colliculus (Figure 4, Dataset 2, Green and Brown). We found that the level of offset information retrieved is the same for both recording modalities in IC. This shows that when sufficient data is acquired in 2P imaging to match the overall information level and compensate for lower SNR, then the information level for offsets also matches.

The reviewer suggests down-sampling 2P imaging data to the number of neurons recorded with electrophysiology. This approach does not yield a fair comparison due to the lower SNR of two-photon imaging data. As we have shown in Bagur et al. Fig. S5A, reproduced below, downsampling decreases retrievable information as expected from lower SNR. Subsampling would eventually be meaningful if we sub-sample two-photon imaging neurons that have the same SNR as electrophysiology neurons. This is a complex procedure that has not been applied in any other study, which we think is beyond the scope of this study and does not add much to the arguments presented above.

Bagur et al. Figure S5A. *Decoding accuracy for spatio-temporal and spatial codes in each area with varying numbers of sub-selected neurons.*

2. Dataset Differences and Variability:The manuscript highlights differences between Dataset 1 and Dataset 2, particularly in the prevalence of combined ON E, sustained E, and OFF E responses and the distribution of offset response fractions (Fig. 3). While the authors attribute these differences to higher variability in Dataset 1, they do not explicitly quantify or visualize this variability. For example:1.The broader distribution of offset response fractions in Dataset 1 cortex (Fig. 3D) suggests higher variability, but the authors should clarify how this variability was measured (e.g., inter-trial correlations, response consistency).2.Differences in stimulus properties, recording conditions, or neural population sampling between the two datasets could also contribute to the observed differences. The authors should discuss these factors in more detail.

We have now provided in Figure 1C a measure of variability based on intertrial correlations for dataset 1, the same measure is found in the now published Bagur et al. 2025 article in Fig. 5A, and this is now mentioned in the manuscript. The plots are reproduced below and show lower reproducibility for 2P imaging than for electrophysiology. Reproducibility values are in the same range for biological repeats (AC and IC), unlike stated in the previous manuscript.

Reproducibility dataset 1 (Fig. 1C):

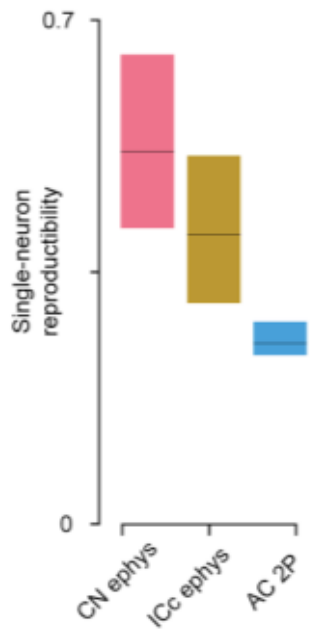

Reproducibility dataset 2

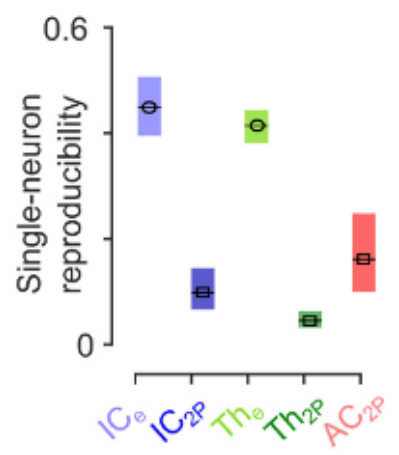

We now mention these measures explicitly in the text in the first paragraph of the results.

Because we have largely changed the statistical approach to detect single cell properties, the details of the analyses reported in Fig. 3 have also changed. Here is the text providing our main conclusions including the discussion of the reason why there are discrepancies between biological repeats.

We therefore plotted in **Fig. 3E** the fractions of all possible combinations of an offset response (either positive, OFF E, or negative, OFF I) with onset (ON E, ON I or none) and sustained (E, I or none) responses. Note that this analysis is done response-by-response and not neuron-by-neuron, and that the fractions are given with respect to the total number of responses with a significant offset response. We observed a fraction of about 40 to 50% pure positive offset responses (OFF E) in the two-photon imaging datasets (**Fig. 3E**), while in the electrophysiology datasets, this fraction was down to 20 to 40%, with variable results across datasets (e.g. IC ephys - dataset 1: 34,58% and dataset 2: 16,97%, **Fig. 3E**). Consistent with our sample data plots of **Fig. 2B&C**, the fraction of pure negative offset was non-negligible (~ 10 to 25% across datasets). The most prominent responses combined with excitatory offsets were sustained excitatory or inhibitory responses (Sustained E OFF E or Sustained I OFF E, **Fig. 3E**), or excitatory responses both in the onset and sustained phases (ON E Sustained E OFF E, **Fig. 3E**) or inhibitory responses both in the onset and sustained phases (ON I Sustained I OFF E, **Fig. 3E**). However, inhibitory responses before the offset were only detected in electrophysiology datasets (**Fig. 3E**). The most prominent responses combined with inhibitory offsets are monophasic onset and sustained responses, either inhibitory or excitatory (ON I Sustained I OFF E, ON E Sustained E OFF I, **Fig. 3E**). However these combinations were also only detected in electrophysiology data, and yet again with some variability, across the IC replicate which could be due to differences in stimulus properties, recording conditions, or neural population sampling (**Fig. 3E**). Altogether, this analysis of combined response type distributions highlighted clear biases across recording modalities likely related to the higher variability of calcium responses, and to their lesser sensitivity to inhibitory response (but note that many inhibitory offset responses were detected with calcium imaging). Beyond this pertaining issue, the analysis shows that across the auditory system offsets are often combined with earlier responses.

3. Fig 3C. Discrepancy in Negative Offset Responses: This is an interesting observation, where the authors provide a reasonable explanation for the discrepancy in negative offset responses (OFF I) between calcium imaging and electrophysiology data, linking it to differences in recording modalities. However, they could strengthen this explanation by: 1. Providing quantitative evidence of the differences in variability between calcium imaging and electrophysiology.2. Discussing whether the discrepancy might also reflect genuine differences in neural processing (e.g., more inhibitory activity in cortical circuits compared to subcortical regions).

1. We have now provided in Figure 1C a measure of variability based on intertrial correlations for dataset 1, the same measure is found in the now published Bagur et al. 2025 article in Fig. 5A, and this is now mentioned in the manuscript.

2. We observe based on IC data that strong differences in response type distribution reflect the recording techniques and the larger amount of noise in two-photon calcium imaging. Hence we did not discuss if the differences could be genuine.

4. The quality and clarity of the figures are generally good, but there is room for enhancement to improve their visual presentation. For instance, the contours in Figure 3C

are nearly invisible. In Figure 2, the heatmaps would benefit from clearer labels and a more detailed legend to assist readers in interpreting the data. Additionally, adjusting the c-axis would better highlight the evoked and suppressed responses. In Figure 3D, is the y-axis label correct? Should it not be the proportional of neurons instead of proportion of stimuli?

We polished the figures. In figure 3D the correct label is ‘fraction of stimulus-neuron pairs’.

5. Terminology: The authors used positive/negative response terms, which are less used, and instead, 'excited' (E) and 'inhibited' (I) are more intuitive and align with the legends in the figures. Using these terms consistently throughout the manuscript would improve clarity and make it easier for readers to interpret the results.

We replaced these terms where needed with inhibitory and excitatory.

6. The introduction lacks more comprehensive review about offset responses and their importance in gap detection (DOI: 10.1093/cercor/bhaa044), perceptual grouping(doi: https://doi.org/10.7551/mitpress/1486.001.0001), noise rejection (doi:10.1007/BF00696152), and their role in prediction-error signaling (omission responses, https://doi.org/10.1016/j.cub.2023.06.013). This would provide a broader context for the study and highlight the importance of offset responses in auditory processing. Additionally, the authors could discuss how their findings relate to the observation that omission responses are primarily cortical (doi:10.1016/j.neuroscience.2016.09.011), suggesting a potential link between offset responses and predictive coding.

We edited the introduction and added the suggested themes and citations, except the last one which we feel requires to stretch the text beyond parsimonious discussion.

Dear Dr Bathellier,

Re: JP-RP-2025-288416R1 "Sound offset responses become highly informative in the auditory cortex" by Charly Lamothe, Sophie Bagur, Etienne Gosselin, and Brice Bathellier

Thank you for submitting your manuscript to The Journal of Physiology. It has been assessed by a Reviewing Editor and by 2 expert referees and we are pleased to tell you that it is acceptable for publication following satisfactory revision.

The referee reports are copied at the end of this email.

Please address all the points raised and incorporate all requested revisions or explain in your Response to Referees why a change has not been made. We hope you will find the comments helpful and that you will be able to return your revised manuscript within 4 weeks. If you require longer than this, please contact journal staff: jp@physoc.org.

Your revised manuscript should be submitted online using the link in your Author Tasks Link Not Available. This link is accessible via your account as Corresponding Author; it is not available to your co-authors. If this presents a problem, please contact journal staff (jp@physoc.org). Image files from the previous version are retained on the system. Please ensure you replace or remove any files that are being revised.

If you do not wish to submit a revised version of your manuscript, you must inform our journal staff (jp@physoc.org) or reply to this email to request withdrawal. Please note that a manuscript must be formally withdrawn from the peer review process at one journal before it may be submitted to another journal.

TRANSPARENT PEER REVIEW POLICY: To improve the transparency of its peer review process The Journal of Physiology publishes online, as supporting information, the peer review history of all articles accepted for publication. Readers will have access to decision letters, including Editors' comments and referee reports, for each version of the manuscript, as well as any author responses to peer review comments. Referees can decide whether or not they wish to be named on the peer review history document.

ABSTRACT FIGURES: Authors are expected to use The Journal's premium BioRender account to create/redraw their Abstract Figures. Information on how to access this account is here:
https://physoc.onlinelibrary.wiley.com/journal/14697793/biorender-access.

This will enable Authors to create and download high-resolution figures. If authors have used the free BioRender service, they can use the instructions provided in the link above to download a high-resolution version suitable for publication.
The link provided should only be used for the purposes of this submission. Authors will be charged for figures created on this account if they are not related to this manuscript submission.

LANGUAGE EDITING AND SUPPORT FOR PUBLICATION: If you would like help with English language editing, or other article preparation support, Wiley Editing Services offers expert help, including English Language Editing, as well as translation, manuscript formatting, and figure formatting at www.wileyauthors.com/eeo/preparation. You can also find resources for Preparing Your Article for general guidance about writing and preparing your manuscript at www.wileyauthors.com/eeo/prepresources.

RIGOUR AND REPRODUCIBILITY: We recommend authors consult The Journal's Rigour and Reproducibility requirements to ensure that their manuscript is suitable for publication: https://physoc.onlinelibrary.wiley.com/pb-assets/hub-assets/physoc/documents/TJP-Rigour-and-Reproducibility-Requirements.pdf

REVISION CHECKLIST:
Check that your Methods section conforms to journal policy: https://jp.msubmit.net/cgi-bin/main.plex?form_type=display_requirements#methods

Check that data presented conforms to the statistics policy: https://jp.msubmit.net/cgi-bin/main.plex?form_type=display_requirements#statistics

Upload a full Response to Referees file. To create your 'Response to Referees' copy all the reports, including any comments from the Senior and Reviewing Editors, into a Microsoft Word, or similar, file and respond to each point, using font or background colour to distinguish comments and responses and upload as the required file type.

Please upload two versions of your manuscript text: one with all relevant changes highlighted and one clean version with no changes tracked. The manuscript file should include all tables and figure legends, but each figure/graph should be uploaded as separate, high-resolution files. The journal is now integrated with Wiley's Image Checking service. For further details, see: https://www.wiley.com/en-us/network/publishing/research-publishing/trending-stories/upholding-image-integrity-wileys-image-screening-service

You may also upload:

- 'Potential Cover Art' for consideration as the issue's cover image
- Appropriate Supporting Information (Video, audio or data set: see https://jp.msubmit.net/cgi-bin/main.plex?form_type=display_requirements#supp).

We look forward to receiving your revised submission.

If you have any queries, please reply to this email and we will be pleased to advise.

Yours sincerely,

Vaughan Macefield
Senior Editor
The Journal of Physiology

REQUIRED ITEMS

- You must start the Methods section with a paragraph headed Ethical approval (https://jp.msubmit.net/cgi-bin/main.plex?form_type=display_requirements#methods).

Research must comply with The Journal's policies regarding animal experiments (https://physoc.onlinelibrary.wiley.com/hub/animal-experiments) and adherence to these policies must be stated in the manuscript.

Authors should confirm in their Methods section that their experiments were carried out according to the guidelines laid down by their institution's animal welfare committee, including an ethics approval reference number. The Methods section must contain a statement about access to food, water and housing, details of the anaesthetic regime: anaesthetic used, dose and route of administration, and method of killing the experimental animals.

- Please include an Abstract Figure file, ***as well as the Figure Legend text within the main article file*** (we seem to be missing the legend). The Abstract Figure is a piece of artwork designed to give readers an immediate understanding of the research and should summarise the main conclusions. If possible, the image should be easily 'readable' from left to right or top to bottom. It should show the physiological relevance of the manuscript so readers can assess the importance and content of its findings. Abstract Figures should not merely recapitulate other figures in the manuscript. Please try to keep the diagram as simple as possible and without superfluous information that may distract from the main conclusion(s). Abstract Figures must be provided by authors no later than the revised manuscript stage and should be uploaded as a separate file during online submission labelled as File Type 'Abstract Figure'. Please also ensure that you include the figure legend in the main article file. All Abstract Figures should be created using BioRender. Authors should use The Journal's premium BioRender account to export high-resolution images. Details on how to use and access the premium account are included as part of this email.

EDITOR COMMENTS

Reviewing Editor:

Both our expert reviewers were satisfied with the provided revisions. However, despite the revisions there is an unacceptable number of typos and language inconsistencies in the manuscript. Please go through the manuscript again word by word and make sure the language matches the quality of the data.

Please check that your article complies with all aspects of our animal ethics policy.

Please provide a legend to accompany your abstract figure.

Senior Editor:

Thank you for submitting your revised manuscript to the Journal of Physiology. While the reviewers and Reviewing Editor are largely satisfied, you will need to thoroughly attend to the typographical errors throughout the manuscript before we can consider this further. I look forward to receiving a revised version in due course.

REFEREE COMMENTS

Referee #1:

The article needs to be carefully edited because it still has a lot of typos, but the statistics are now acceptable, in my opinion.

Referee #2:

The authors have satisfactorily addressed my concerns regarding methodological rigor and data interpretation. Their revisions have strengthened the manuscript through improved quantification of signal variability, refined statistical approaches, and clearer presentation of results. The expanded introduction now properly situates this work within the field. I find their responses sound and recommend publication in its current form.

END OF COMMENTS

Dear Editors,

Following your request the resubmitted manuscript:

- Includes a statement that all aspects of our experiments comply with the Journal's ethical policies, as we have checked. This statement is included in the Ethical Approval paragraph introducing the method section and copied below:

Ethical approval

All experimental and surgical procedures were carried out in accordance with the European guidelines for animal experiments and were validated by the French Ethical Committee #89 (authorization APAFIS#27040-2020090316536717 v1). All experimental and surgical procedures strictly comply with The Journal of Physiology's policies regarding animal experiments, including housing, anesthesia and terminal procedures as described below.

- Includes a caption for the Abstract Figure
- Was corrected by a native English speaker. We apologize for the typos found in the previous version of the manuscript.

Dear Dr Bathellier,

Re: JP-RP-2025-288416R2 "Sound offset responses become highly informative in the auditory cortex" by Charly Lamothe, Sophie Bagur, Etienne Gosselin, and Brice Bathellier

We are pleased to tell you that your paper has been accepted for publication in The Journal of Physiology.

The last Word (or similar) version of the manuscript provided will be used by the Production Editor to prepare your proof. When this is ready you will receive an email containing a link to Wiley's Online Proofing System. The proof should be thoroughly checked and corrected as promptly as possible.

Authors should note that it is too late at this point to offer corrections prior to proofreading. Major corrections at proof stage, such as changes to figures, will be referred to the Editors for approval before they can be incorporated. Only minor changes, such as to style and consistency, should be made at proof stage. Changes that need to be made after proof stage will usually require a formal correction notice.

All queries at proof stage should be sent to: TJP@wiley.com.

Following copyediting and typesetting the accepted version will be published online.

Yours sincerely,

Vaughan Macefield
Senior Editor
The Journal of Physiology

IMPORTANT POINTS TO NOTE FOLLOWING YOUR PAPER'S ACCEPTANCE:

If you would like to receive our 'Research Roundup', a monthly newsletter highlighting the cutting-edge research published in The Physiological Society's family of journals (The Journal of Physiology, Experimental Physiology, Physiological Reports, The Journal of Nutritional Physiology and The Journal of Precision Medicine: Health and Disease), please click this link, fill in your name and email address and select 'Research Roundup':
https://www.physoc.org/journals-and-media/membernews

• TRANSPARENT PEER REVIEW POLICY: To improve the transparency of its peer review process, The Journal of Physiology publishes online as supporting information the peer review history of all articles accepted for publication. Readers will have access to decision letters, including Editors' comments and referee reports, for each version of the manuscript as well as any author responses to peer review comments. Referees can decide whether or not they wish to be named on the peer review history document.

• You can help your research get the attention it deserves! Check out Wiley's free Promotion Guide for best-practice recommendations for promoting your work at: www.wileyauthors.com/eeo/guide. You can learn more about Wiley Editing Services which offers professional video, design, and writing services to create shareable video abstracts, infographics, conference posters, lay summaries, and research news stories for your research at: www.wileyauthors.com/eeo/promotion.

• IMPORTANT NOTICE ABOUT OPEN ACCESS: To assist authors whose funding agencies mandate public access to published research findings sooner than 12 months after publication, The Journal of Physiology allows authors to pay an Open Access (OA) fee to have their papers made freely available immediately on publication.

The Corresponding Author will receive an email from Wiley with details on how to register or log-in to Wiley Authors Services where you will be able to place an order.

• You can check if your funder or institution has a Wiley Open Access Account here: https://authorservices.wiley.com/author-resources/Journal-Authors/licensing-and-open-access/open-access/author-compliance-tool.html.

EDITOR COMMENTS

Reviewing Editor:

Thank you for the thorough revision.

Senior Editor:

Thank you for attending to the remaining comments. I am pleased to report your manuscript is now acceptable for publication.
